# A local water molecular-heating strategy for near-infrared long-lifetime imaging-guided photothermal therapy of glioblastoma

**DOI:** 10.1038/s41467-023-38451-3

**Published:** 2023-05-13

**Authors:** Dongkyu Kang, Hyung Shik Kim, Soohyun Han, Yeonju Lee, Young-Pil Kim, Dong Yun Lee, Joonseok Lee

**Affiliations:** 1 Department of Chemistry, Hanyang University, Seoul, 04763 Republic of Korea; 2 Department of Bioengineering, College of Engineering, and BK FOUR Biopharmaceutical Innovation Leader for Education and Research Group, Hanyang University, Seoul, 04763 Republic of Korea; 3 Department of HY-KIST Bio-Convergence, Hanyang University, Seoul, 04763 Republic of Korea; 4 Department of Life Science, Hanyang University, Seoul, 04763 Republic of Korea; 5 Institute of Nano Science and Technology (INST), Hanyang University, Seoul, 04763 Republic of Korea; 6 Research Institute for Convergence of Basic Sciences, Hanyang University, Seoul, 04763 Republic of Korea; 7 Institute for Bioengineering and Biopharmaceutical Research (IBBR), Hanyang University, Seoul, 04763 Republic of Korea; 8 Elixir Pharmatech Inc., Seoul, 07463 Republic of Korea

**Keywords:** Fluorescence imaging, Optical imaging, Nanoparticles, Cancer imaging

## Abstract

Owing to the strong absorption of water in the near-infrared (NIR) region near 1.0 μm, this wavelength is considered unsuitable as an imaging and analytical signal in biological environments. However, 1.0 μm NIR can be converted into heat and used as a local water-molecular heating strategy for the photothermal therapy of biological tissues. Herein, we describe a Nd-Yb co-doped nanomaterial (water-heating nanoparticles (NPs)) as strong 1.0 μm emissive NPs to target the absorption band of water. Furthermore, introducing Tm ions into the water-heating NPs improve the NIR lifetime, enabling the development of a NIR imaging-guided water-heating probe (water-heating NIR NPs). In the glioblastoma multiforme male mouse model, tumor-targeted water-heating NIR NPs reduce the tumor volume by 78.9% in the presence of high-resolution intracranial NIR long-lifetime imaging. Hence, water-heating NIR NPs can be used as a promising nanomaterial for imaging and photothermal ablation in deep-tissue-bearing tumor therapy.

## Abstract (web summary)

Neodymium (Nd)-doped nanoparticles have been described for imaging-guided photothermal therapy. Here the authors design a Nd-Yb co-doped nanomaterial as nearinfrared long-lifetime imaging-guided waterheating probe, showing photothermal ablation in a glioblastoma pre-clinical mode

## Introduction

Lanthanide-doped upconversion nanoparticles (UCNPs), which absorb low-energy photons and emit high-energy photons, have been recognized as a type of bioimaging probe. Particularly, Yb-Tm co-doped NaYF_4_ has been extensively studied because near-infrared (NIR) light is more suitable for bioimaging applications instead of visible regions^1–3^. Recently, as emerging theranostic nanoparticles (NPs), neodymium (Nd)-doped NPs, such as NaNdF_4_, LaF_3_:Nd^3+^, and NdVO_4_ materials, have demonstrated good photostability and multifunctionality for imaging-guided photothermal therapy (PTT)^4–7^. These NPs generate heat by cross-relaxation (CR), a phenomenon that occurs between Nd^3+^ ions themselves or other neighboring ions in ladder-like energy states. This cascade energy transfer in Nd^3+^ ions can generate heat by an interionic interaction between the states of (^4^F_3/2_, ^4^I_15/2_) ↔ (^4^I_15/2_, ^4^I_9/2_), and its photothermal performance is determined by the doping concentrations of Nd^3+^ since CR mainly depends on the distance between Nd^3+^ ions^8,9^. However, the photothermal performance of Nd-doped nanomaterials is not sufficient to achieve the threshold temperature for thermal ablation of tumors even with highly Nd-doped photothermal agents because their absorption coefficient is small and narrow around 800 nm (their photothermal conversion efficiency was less than 10%)^8,10^. To overcome this limitation, NaNdF_4_ NPs have been additionally decorated with heterogeneous materials such as Prussian blue, CuS, MnO_2_, and carbon^8,11^. Nevertheless, heterogeneous decoration could lead to an adverse effect on the optical properties such as absorption ability and luminescence efficiency.

In this context, we developed Nd-sensitized 1.0 μm emissive core@shell NPs (NaYF_4_:50%Yb@NaYF_4_:40%Nd,20%Yb, denoted as water-heating NPs) as a local water-molecular heating material. In particular, the strong energy absorption coefficient derived from the overtones and the stretching vibrations of O–H oscillators matches the emission spectrum of water-heating NPs. Therefore, the energy from the luminescence at approximately 1.0 μm can be non-radiatively transferred to stretching vibrations of nearby coordinated solvent molecules^12^. The 1.0 μm luminescence-induced photothermal conversion efficiency was 23.3% in aqueous solution, which is ~3 times higher than that of the conventional CR-induced photothermal effect.

Furthermore, to design a multifunctional single NP as a NIR imaging-guided PTT agent, we added 2% Tm ions into the water-heating NPs (NaYF_4_:50%Yb2%Tm@NaYF_4_:40%Nd,20%Yb, denoted as water-heating NIR NPs).

**Fig. 1:**
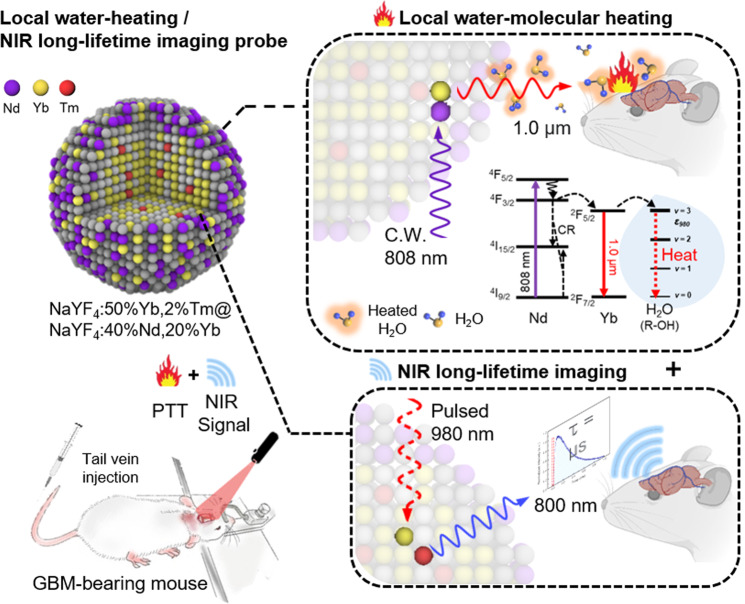
Schematic illustrations of Local water-heating NIR long-lifetime imaging probe. Schematic illustration of a multifunctional NP (NaYF_4_:50%Yb,2%Tm@NaYF_4_:40%Nd,20%Yb) enabling (top) 1.0-µm-light-induced local water-molecular heating under an 808 nm CW laser and the corresponding energy diagram. (bottom) NIR long-lifetime imaging under a 980 nm pulsed laser.

Multifunctional nanomaterial-based therapeutic methods are an area of active research^13^. Among these methods, real-time imaging-guided PTT is regarded as a promising strategy owing to its minimal invasiveness and the convenience of on-site manipulation^14^. However, the practical use of conventional intensity-based image measurement is still challenging due to the perturbed luminescence signal by the relative penetration depth, laser-induced autofluorescence, and background interference^15,16^. Hence, we applied this NIR probe to the time-resolved luminescence imaging technology (lifetime imaging). The NIR luminescence signal from the upconversion has a microsecond lifetime that is effectively distinguished from the autofluorescence and background noise of biological molecules with a nanosecond lifetime by exerting delay time^15^. Herein, we report the design of homogeneously structured core@shell NPs (water-heating NIR NPs), NIR long-lifetime imaging of glioblastoma multiforme (GBM) with targeted water-heating NIR NPs, and the selective destruction of GBM induced by local water-molecular heating as a PTT strategy (Fig. 1).

The biological application of water-heating NIR NPs was examined as a NIR long-lifetime imaging-guided PTT in the orthotopic GBM-bearing mouse model. In general, brain tumors have various barriers such as the scalp, skull, and surrounding normal brain tissue, making light penetration to the tissue difficult and reaching the GBM lesion. An anti-CD133 antibody capable of interacting with a transmembrane protein (CD133) and preferentially localizing in the membrane of GBM cells was conjugated to the water-heating NIR NPs (Ab-NPs) to circumvent the non-specific distribution of water-heating NIR NPs. Hence, Ab-NPs targeted GBM lesions 2.4 times compared with the unconjugated water-heating NIR NPs, and NIR long-lifetime imaging and PTT were successfully achieved by 980 nm pulsed laser and 808 nm continuous wave (CW) laser irradiation, respectively. To the best of our knowledge, this is the first time that 1.0 μm luminescence emissive water-heating NIR NPs have been administered as a water-heating material to eliminate GBM tumor cells. Systematic in vitro and in vivo PTT and NIR long-lifetime imaging of intracranial GBM were performed to evaluate their brain tumor targeting, deep-tissue imaging, and anti-tumor therapeutic efficacy. This multifunctional material, with negligible in vivo toxicity as confirmed by the histological examination, holds great potential in future NIR long-lifetime imaging-guided PTT.

## Results

### Preparation and characterization of local water-molecular heating NPs

NaYF_4_:10%Nd,4%Yb NPs (water-heating core) were synthesized for a 1.0 µm luminescence emission under 808 nm excitation via a thermal decomposition method^17^. The morphology of the synthesized water-heating core was uniform and spherical with an average NP size of 18.5 ± 1.7 nm (Supplementary Fig. S1a). The X-ray diffraction (XRD) patterns show that NPs consist of pure hexagonal phase NaYF_4_ (Supplementary Fig. S1b)_._

**Fig. 2:**
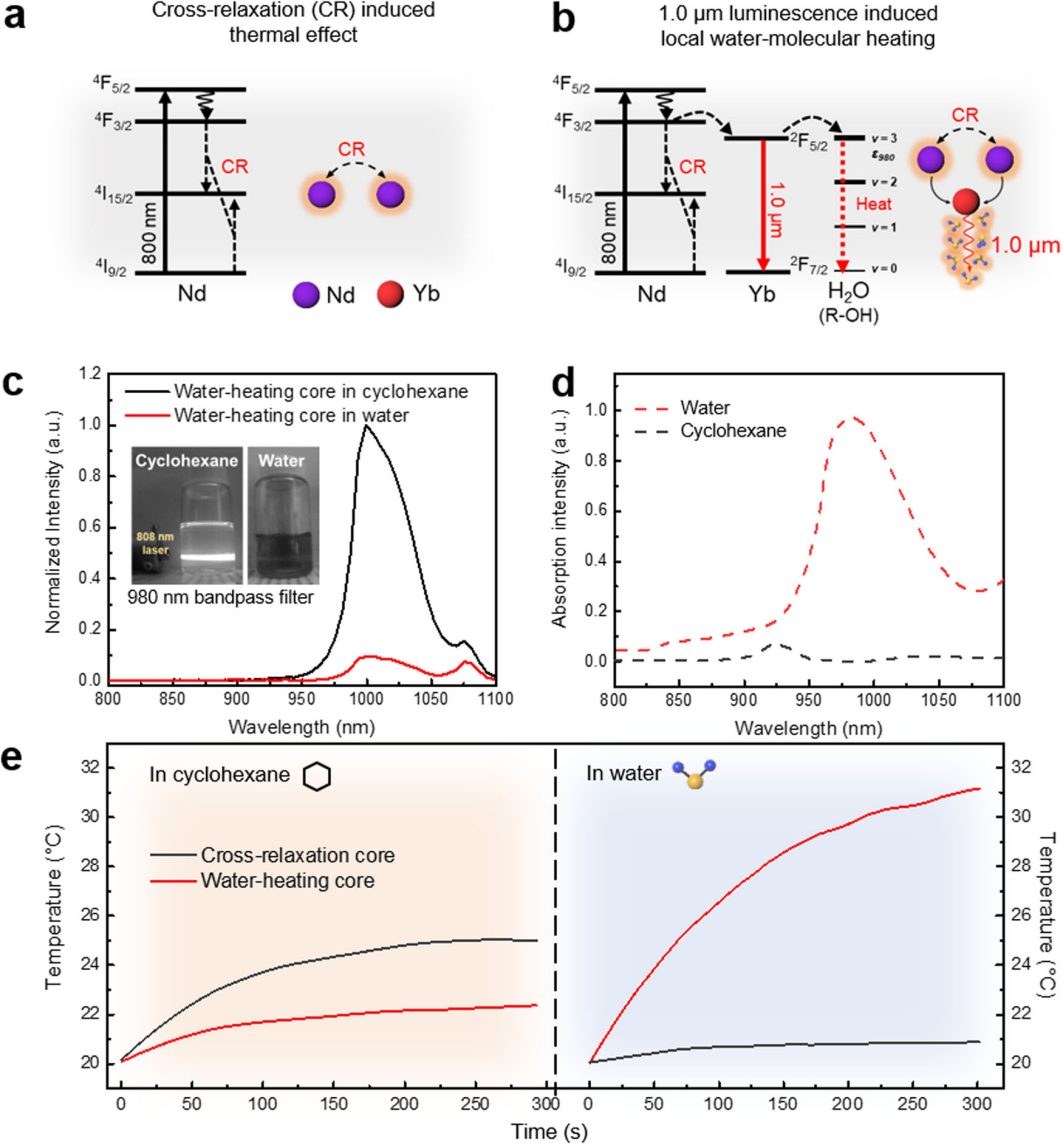
Characterization of the water-heating core. **a** Illustrations of CR-induced thermal effect in Nd-doped NPs and (**b**) 1.0-μm-luminescence-induced water-heating effect in aqueous solution. **c** Diminished 1.0 μm emission spectrum of the water-heating core in cyclohexane and a water solution at a power density of 0.1 W/cm^2^. Inset: optical images of the water-heating cores in cyclohexane and water; a.u., arbitrary units. **d** NIR absorbance spectrum of cyclohexane and water. **e** Comparison of photothermal effects between the water-heating core (red) and CR-core (black) under the excitation of an 808 nm laser at a power density of (left) 0.5 W/cm^2^ in cyclohexane and (right) 2.0 W/cm^2^ in water. Source data are provided as a Source Data file.

To demonstrate the water-heating effect of the 1.0 µm luminescence emissive water-heating core, we compared it with NaYF_4_:10%Nd NPs (CR-core), which exhibits the photothermal effect only by conventional CR without 1.0 µm luminescence. As shown in Fig. 2a, CR-induced heat was generated from the interionic interaction between the states of (^4^F_3/2_, ^4^I_15/2_) and (^4^I_15/2_, ^4^I_9/2_) under 808 nm excitation in both Nd^3+^-doped NPs (water-heating core and CR-core)^18^. We, therefore, expected that the 10% Nd^3+^ doping concentration of the water-heating core and CR-core showed a similar level of photothermal performance in the cyclohexane solution because their heat generation was only attributed to CR. The main difference between the water-heating core and CR-core is the presence of Yb^3+^ ions in the water-heating core, which plays a major role as a 1.0 µm luminescent sensitizer to heat water molecules (Fig. 2b).

Water-soluble NPs were subsequently prepared via a surface ligand exchange of the oleic acid-capped hydrophobic NPs with dopamine hydrochloride (dopamine-coated NPs) to investigate the photothermal effect in an aqueous solution. Fourier transform infrared (FT-IR) spectra showed that the surface ligand of oleic acids was replaced successfully with the amine ligand (Supplementary Fig. S2)^3,19^. As a result of water-solubilization, the wavelength of around 1.0 μm emitted from the water-heating core was strongly absorbed by distilled water (Fig. 2c, d). Figure 2e shows the temperature rise in CR-core and water-heating core under 808 nm CW laser excitation. The photothermal performance of the CR-core and water-heating core in cyclohexane solution was similar, resulting in a temperature rise of 24.2 and 21.8 °C, respectively. Contrary to the cyclohexane solution, the water-heating core showed a much higher temperature increase (31.1 °C) than that of the CR-core (20.9 °C) in the aqueous solution. Hence, the water-heating core provides photothermal efficiency in aqueous solutions by heating water molecules using 1.0 μm luminescence. However, the photothermal performance of the current water-heating core (31.1 °C) is insufficient for the thermal ablation of tumors because tumor ablation by PTT generally requires a temperature rise above 45 °C^20^.

We further developed and optimized the core@shell structured Nd-Yb co-doped NPs (noted: NaYF_4_:x%Yb@NaYF_4_:20%Yb,y%Nd) to maximize the heating effect from 1.0 μm luminescence. These core@shell NPs have a significant advantage in that they can reduce the deleterious energy back-transfer since the Nd^3+^ ions (sensitizers) were well separated from Yb^3+^ (activators) by the core@shell structure^21^. As a result of optimization (Fig. 2a, b and Supplementary Figs. S3, S4), we confirmed 1.0 µm luminescence intensity of NaYF_4_:50%Yb@NaYF_4_:20%Yb,40%Nd NPs (water-heating NPs, core@shell NPs) is 2.17-times higher than that of the core NPs. Resultantly, the Nd^3+^ concentration in the shell layer was optimized at a higher value of 40 mol% compared with the CR-core (Nd 10 mol%). Hence, the design of core@shell helped both higher luminescence intensity and CR by achieving higher doping concentrations and minimizing energy back-transfer. The synthesized water-heating NPs were monodispersed and spherical with an average particle size of 50.1 ± 1.8 nm. NaYF_4_@NaYF_4_:40%Nd core@shell NPs (CR NPs) were synthesized as a counterpart of water-heating NPs, which are like CR-core and exhibit the photothermal effect only by CR without the 1.0 µm luminescence (Supplementary Fig. S5a). The XRD patterns of CR NPs are consistent with the hexagonal NaYF_4_ (Supplementary Fig. S5b). The surface of two NPs (water-heating NPs and CR NPs) was modified through the ligand ionization exchange method for the same purpose in the comparative experiment of water-heating core and CR-core (Supplementary Fig. S6)^3,19^.

**Fig. 3:**
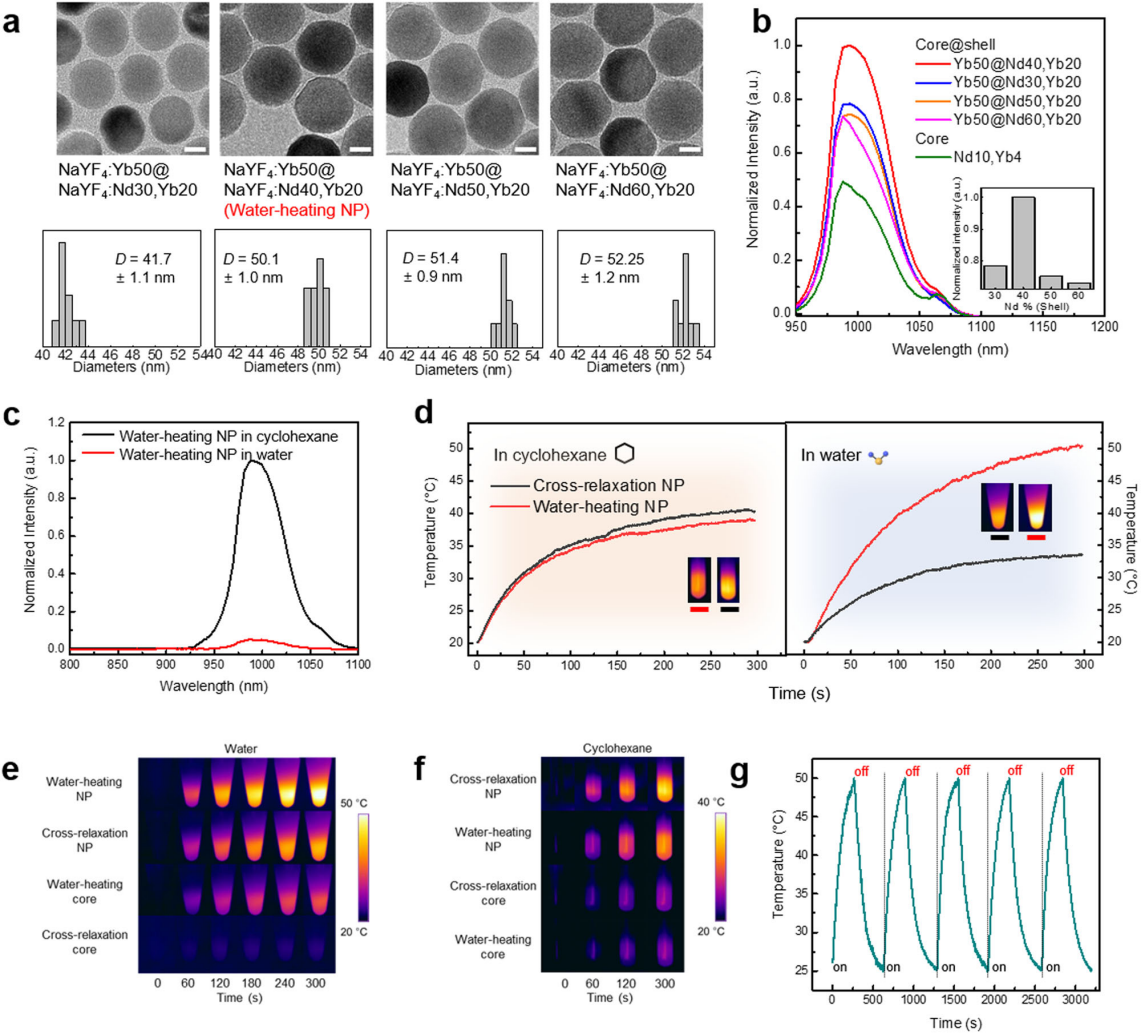
Characterization of water-heating NPs. **a** TEM images and size distributions of Nd–Yb co-doped core@shell NPs (41.7 ± 1.1, 50.1 ± 1.0, 51.4 ± 0.9 nm, and 52.2 ± 1.2 nm, respectively), scale bar: 20 nm. 3-TEM images of each sample were included for statistical analysis, the results were presented as mean ± standard deviation. **b** 1.0 μm emission spectrum of Nd–Yb co-doped core@shell NPs; inset: corresponding intensities; a.u., arbitrary units. **c** Diminished 1.0 μm emission spectrum of water-heating NP in cyclohexane and water at a power density of 0.1 W/cm^2^. **d** Comparison of photothermal effects between water-heating NPs (red) and CR NPs (black) under the excitation of 808 nm at a power density of (left) 0.5 W/cm^2^ in cyclohexane and (right) 2.0 W/cm^2^ in water solution. Thermal images of the NPs in (**e**) water and (**f**) cyclohexane. **g** Photothermal stability under 808 nm laser irradiation during five on–off cycles. Source data are provided as a Source Data file.

The unparalleled 1.0 μm luminescence of water-heating NPs leads to the high absorption of 1.0 μm light by water, resulting in a 1.0 μm emission reduction of up to 97% in the aqueous solution compared to the cyclohexane solution (Fig. 3c). The temperature rises by the photothermal effects of water-heating NPs and CR NPs were investigated in the cyclohexane and aqueous solution (Figs. 3d, f). In cyclohexane solution, water-heating NPs and CR NPs resulted in a temperature increase of 37.5 °C (Red line) and 38.3 °C (Black line), respectively, under 808 nm laser irradiation. Conversely, in an aqueous solution, water-heating NPs resulted in a much higher temperature increase of approximately 50.4 °C (Red line) while CR NPs resulted in a temperature increase of 34.6 °C (Black line). According to the model reported by Roper et al., the photothermal conversion efficiencies of water-heating NPs and CR NPs were calculated to be 23.3% and 8.2%, respectively, in an aqueous solution (Supplementary Fig. S7)^22^. This result implies that the intense 1.0 μm light was converted into thermal energy by the strong absorption of water. Note that the specific heat capacity of cyclohexane is 1.8 J/kg °C, which is much less than that of water (4.18 J/kg °C)^23^. Cyclohexane thus requires less heat than water to reach the same temperature. Naturally, the photothermal conversion efficiency of the NPs in cyclohexane is higher than that in water. Nevertheless, in the practical use of PTT, NPs should be sufficiently dispersible in water to enable them to target tumor sites. The water-heating NPs also demonstrated significant thermal stability after several cycles of exposure to 808 nm irradiation (Fig. 3g). The temperature profiles of water-heating NPs at different concentrations and power densities are presented in Supplementary Fig. S8. Evidently, the temperature increased with the increasing NP concentration and the increasing laser power density, which indicates that the therapeutic conditions can be designed for the cancer environment.

To investigate the photothermal effect of the ligand, we prepared other water-soluble NPs, such as polyacrylic acid (PAA)-coated NPs and ligand-free NPs^24,25^ (Supplementary Fig. S9). As shown in Supplementary Fig. S9c, all three types of NPs showed the same absorption ability in both the visible and NIR regions, confirming that the ligand-induced photothermal effect was negligible (Supplementary Fig. S9d).

### Preparation and characterization of NIR long-lifetime imaging-guided water-heating photothermal probes

**Fig. 4:**
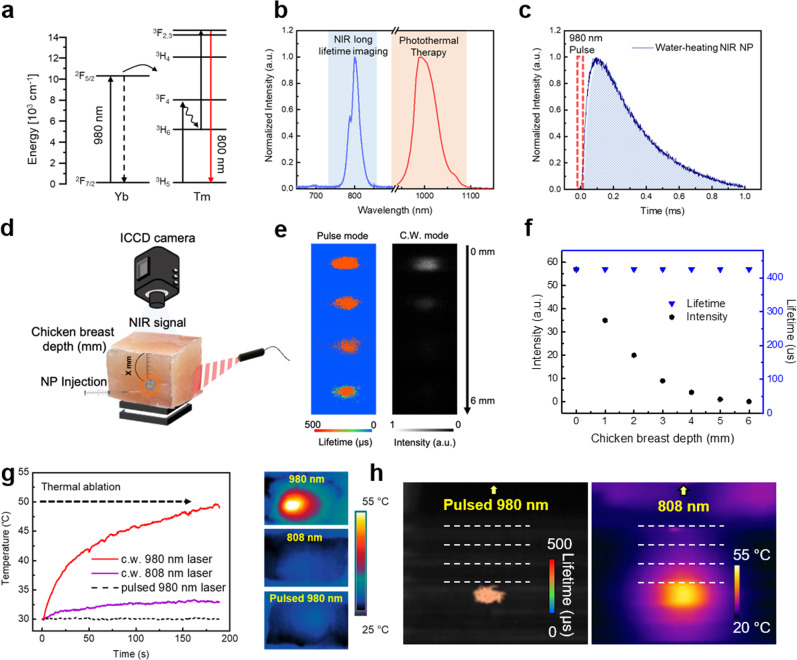
Characterization of the lifetime properties of water-heating NIR NPs. **a** Energy diagram of the upconversion process in water-heating NIR NPs. **b** Dual emission spectrum of the final water-heating NIR NPs: (left) NIR at 800 nm emission spectrum for NIR long lifetime imaging under pulsed 980 nm laser and (right) 1.0 μm luminescence spectrum for PTT under an 808 nm CW laser; a.u., arbitrary units. **c** Lifetime profile of 800 nm emission under 980 nm pulsed excitation, with a period of 0.1 ms and a power density of 1.0 W/cm^2^. **d** Schematic illustration of in vitro chicken breast experimental procedure. **e** Comparison of the lifetime image (left) and conventional intensity-based imaging (right) with increasing depth in the chicken breast (0–6 mm). **f** Normalized intensities and lifetime obtained from the water-heating NIR NP emission. **g** In vitro infrared thermal images of the chicken breast under an 808 nm CW laser (1.5 W/cm^2^), a 980 nm CW laser (1.5 W/cm^2^), and a pulsed 980 nm laser (1.5 W/cm^2^). **h** NIR optical image and thermal image of the intralipid 1% solution under the (left) a pulsed 980 nm laser (1.0 W/cm^2^) and (right) a 808 nm CW laser (1.5 W/cm^2^) in a capillary tube. Source data are provided as a Source Data file.

To give water-heating NPs NIR luminescence property, we added 2% Tm^3+^ ions into the core structure, which has an 800 nm NIR emission spectrum under 980 nm laser excitation (Fig. 4a, b)^26^. As shown in Supplementary Fig. S10a, energy-dispersive spectroscopy (EDS) mapping of NaYF_4_:50%Yb,2%Tm@NaYF_4_:40%Nd,20%Yb (water-heating NIR NPs, which enables both PTT and NIR long-lifetime imaging) confirmed that the lanthanides are well dispersed in water-heating NIR NPs. TEM images of water-heating NIR NPs show similar morphology and size to water-heating NPs with an average size of 49.2 ± 1.7 nm (Supplementary Fig. S10b). Finally, two distinguished emissions were facilitated including 800 nm emission for NIR long-lifetime imaging and 1.0 μm luminescence for photothermal effect under 980 nm pulsed laser and 808 nm CW laser excitation, respectively (Fig. 4b and Supplementary Fig. S10c, S10d). The 1.0 μm emission intensity was maintained while introducing the 2% Tm^3+^ ions (Supplementary Fig. S10e, S10f). In addition, doping Tm^3+^ into the water-heating NPs negligibly influenced the luminescence and photothermal conversion efficiency. As shown in Fig. 4c, their luminescence lifetime profiles were obtained using a function generator, with a period of 0.1 ms. Their lifetime decays were obtained from exponential fitting on a selected region of interest (ROI) of the image and calculated to be 436 ± 3.5 µs in aqueous solution^27^. Moreover, we investigated the change in the luminescence lifetime with increasing temperature. As shown in Supplementary Fig. S11, the lifetime decreased from 441 μs (20 °C) to 419 μs (50 °C) under an extreme temperature change. According to Liu et al.^28^, a temperature increase could change the lifetime because the heat might deactivate Yb ions. However, our experimental method was based on the lifetime-imaging guided PTT. We conducted lifetime imaging with a 980 nm pulsed laser, which does not affect temperature, and then, we implemented PTT with an 808 nm CW laser. Therefore, the lifetime imaging was performed at the same temperature before thermal ablation.

Figure 4d schematically illustrates the chicken breast for in vitro NIR long-lifetime imaging experiments. NIR emission intensities from water-heating NIR NPs are diminished as the chicken breast depth increases (0–6 mm) for traditional intensity-based imaging measurements under CW laser excitation. In contrast, lifetime imaging derived from the decay time recognized their NIR emission against the background signal, with consistent pseudo-colors (Figs. 4e, f). Furthermore, overheating from the 980 nm excitation light may limit the practical in vivo imaging^20^. Thus, we investigated the thermal effects of the pulsed 980 nm laser on overheating compared to the 980 nm CW laser. As a result of the pulsed 980 nm laser irradiation, the temperature of the chicken breast increased by ~3 °C with a period of 0.1 ms and a power density of 1.0 W/cm^2^. However, the irradiation of the 980 nm CW laser increased temperature by more than 50 °C, which leads to the risk of overheating and unwanted thermal effects (Fig. 4g). Therefore, the overheating effect of the pulsed 980 nm laser is negligible as a source of NIR long-lifetime in vivo imaging^29^. Moreover, we investigated the NIR long-lifetime imaging and water-heating effect in 1% intralipid solutions in capillary tubes with a diameter of 1 mm. The 1% intralipid was used as a mimic of biological tissue to confirm the clarity of lifetime imaging and local water-molecular heating in the capillary tube^30^. Water-heating NIR NPs (5 mg/mL) were filled into one capillary tube. The capillary tube was then placed among the other tubes with only 1% intralipid solution. As shown in Fig. 4h, their lifetime profile was calculated to be 432 ± 1.7 µs under excitation of 980 nm pulsed laser and excitation of 808 nm CW laser, and the temperature was increased up to 55 °C with a depth of 4 mm at 1% intralipid solution (Fig. 4h).

### In vitro enhancement of NIR long-lifetime imaging and PTT efficacy by conjugating targeting ligand to water-heating NIR NPs

To further explore the potential for biological application, we validated the NIR long-lifetime imaging and photothermal therapeutic effect of water-heating NIR NPs on GBM cell lines (U87MG). Anti-CD133 monoclonal antibody (anti-CD133 mAb) was conjugated to water-heating NIR NPs to perform NIR long-lifetime imaging-guided PTT after systemic delivery into GBM and denoted as Ab-NPs (anti-CD133 mAb conjugated water-heating NIR NPs) and bare-NPs (absence of anti-CD133 mAb conjugation to water-heating NIR NPs) (Supplementary Fig. S6). To investigate their stability, the Ab-NPs were dispersed in a 1 mM PBS solution and a 10% fetal bovine serum (FBS) solution and stored at 4 °C, and the changes in the luminescence intensity and polydispersity index (PDI) were monitored by dynamic light scattering. As shown in Supplementary Fig. S12, the size of Ab-NP in the 1 mM PBS and 10% FBS solutions did not change obviously during the two-week observation period^31,32^.

**Fig. 5:**
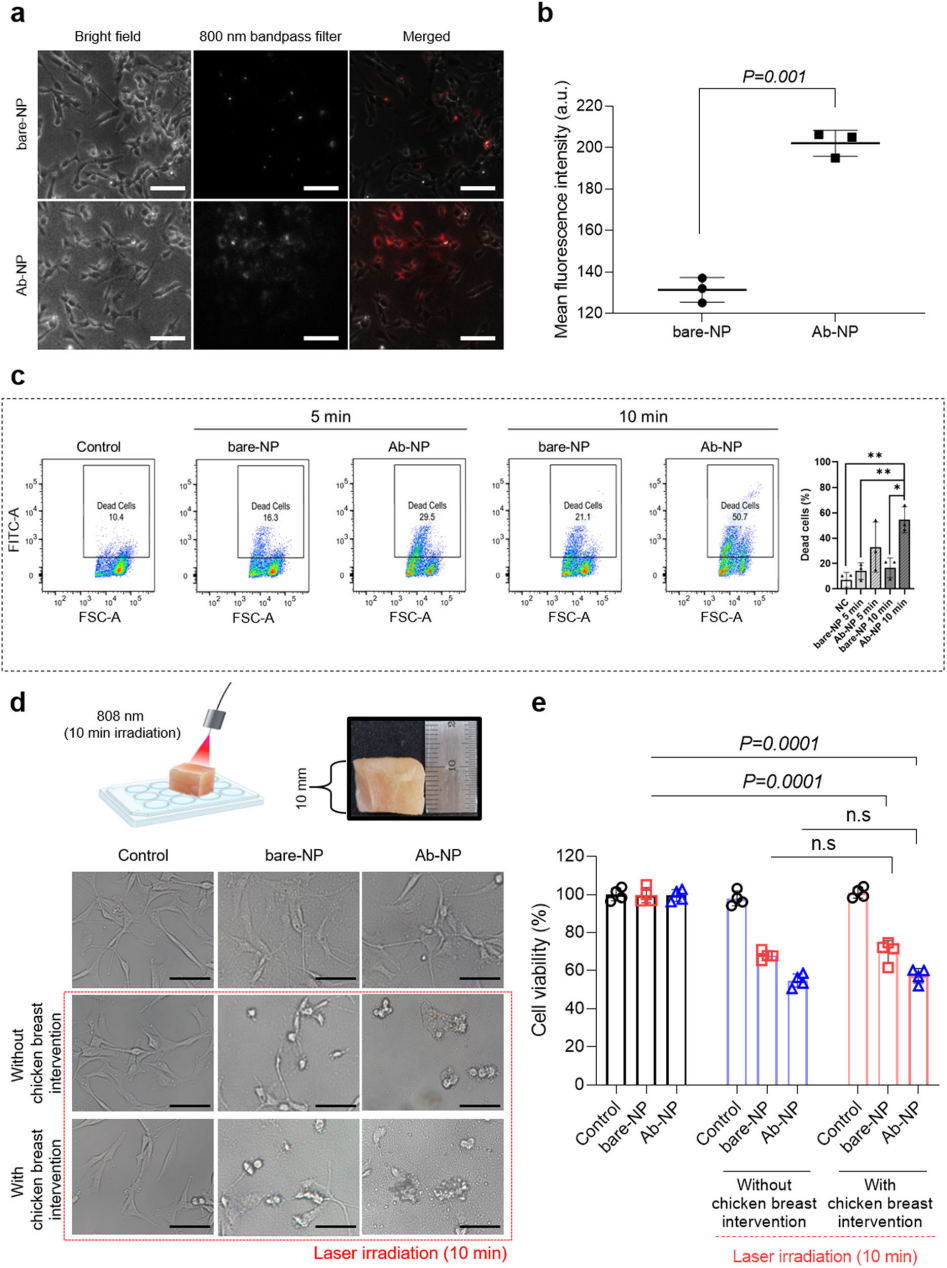
In vitro NIR imaging and PTT effect on the glioma cell (U87MG). **a** Confocal microscopy images of bare- and Ab-NPs on U87MG cells. Scale bars: 100 μm. **b** Quantification of cellular uptake by MFI (a.u.) measurement. Data are expressed as mean ± SEM (*n* = 3 biological independent cells). Two-sided *P* values calculated by Student’s *t* tests. **c** Investigation of PTT efficacy of 5 and 10 min of laser irradiation with bare- and Ab-NPs. Data are expressed as mean ± SEM (*n* = 3). *P < 0.05, **P < 0.01, ***P < 0.001. **d** Schematic of the experimental setup for the evaluation of laser tissue penetration and images of the cell morphology after the respective treatments. Scale bars: 25 μm. **e** Analysis of laser-exposed cell viability with and without tissue intervention. Bare- and Ab-NPs were used at a concentration of 800 μg/mL per well. Data are expressed as mean ± SEM (*n* = 4 biological independent cells). Two-sided *P* values calculated by Student’s *t* tests. Source data are provided as a Source Data file.

Cellular uptakes were compared between the bare-NPs and Ab-NPs by confocal microscopy and quantification analysis (Figs. 5a, b). As a result, the intracellular NIR signal was significantly increased in Ab-NPs compared with bare-NPs even for a short drug incubation time (approximately ~10 min). In vitro experiments were performed for a short period to exclude diffuse absorption of the drug, as there is a possibility of passive diffusion with a long incubation period. Consistent with images of intracellular NIR signal, the amounts of cellular uptakes were 132 ± 5.1 and 195 ± 11.0 (a.u.) in the bare-NPs and Ab-NPs, respectively. This increase in cellular uptake by conjugating anti-CD133 mAb to water-heating NIR NPs further potentiates phototoxicity in laser irradiation. The dead cell population in the Ab-NPs was 18.4% and 47.7% after 5 and 10 min of laser irradiation, respectively, whereas those for bare-NPs did not increase even after 10 min of laser irradiation (Fig. 5c). This indicates the significance of intracellular drug accumulation to exhibit a sufficient PTT effect in GBM. (Supplementary Fig. S13, A representative FACS sequential gating/sorting strategy).

Laser tissue penetration is also considered an important issue for a satisfactory effect of PTT in the GBM. We, therefore, analyzed the laser-exposed cell viability in the existence or absence of chicken breast intervention (~1 cm thickness) to investigate laser tissue penetration with Ab-NPs (Fig. 5d). In the morphological investigation, there was no apparent cellular damage in both the bare-NPs and Ab-NPs treated groups without laser exposure. Conversely, cellular destruction was observed in the laser-applied group via membrane rupture and numerous intracellular bubbles, which evidence hyperthermia via PTT^33^. It was found in both bare-NPs and Ab-NPs, regardless of whether chicken breast was involved. Consistent with the morphology study, cell viability was almost identical in both the presence or absence of chicken breast intervention, indicating that CW laser photon (808 nm, 2.5 W/cm^2^) penetrates 100% through the 1 cm thick tissue and sufficiently induces PTT on GBM cells (Fig. 5e). More specifically, to investigate the correlation between the laser-transmitted energy density and heat generation according to the depth in the chicken breast, each index was measured for chicken breast thicknesses of 0–15 mm (Supplementary Fig. S14). As a result, the power density gradually decreased from 2.5 to 1.5 W/cm^2^ under the interference of the 10 mm chicken breast, but the heat generation was maintained up to 43 °C. This finding corroborates the equivalent cell viability results, regardless of the chicken breast intervention.

### Significant NIR long-lifetime imaging and PTT effects of Ab-NP on the GBM-bearing orthotopic mouse model

**Fig. 6:**
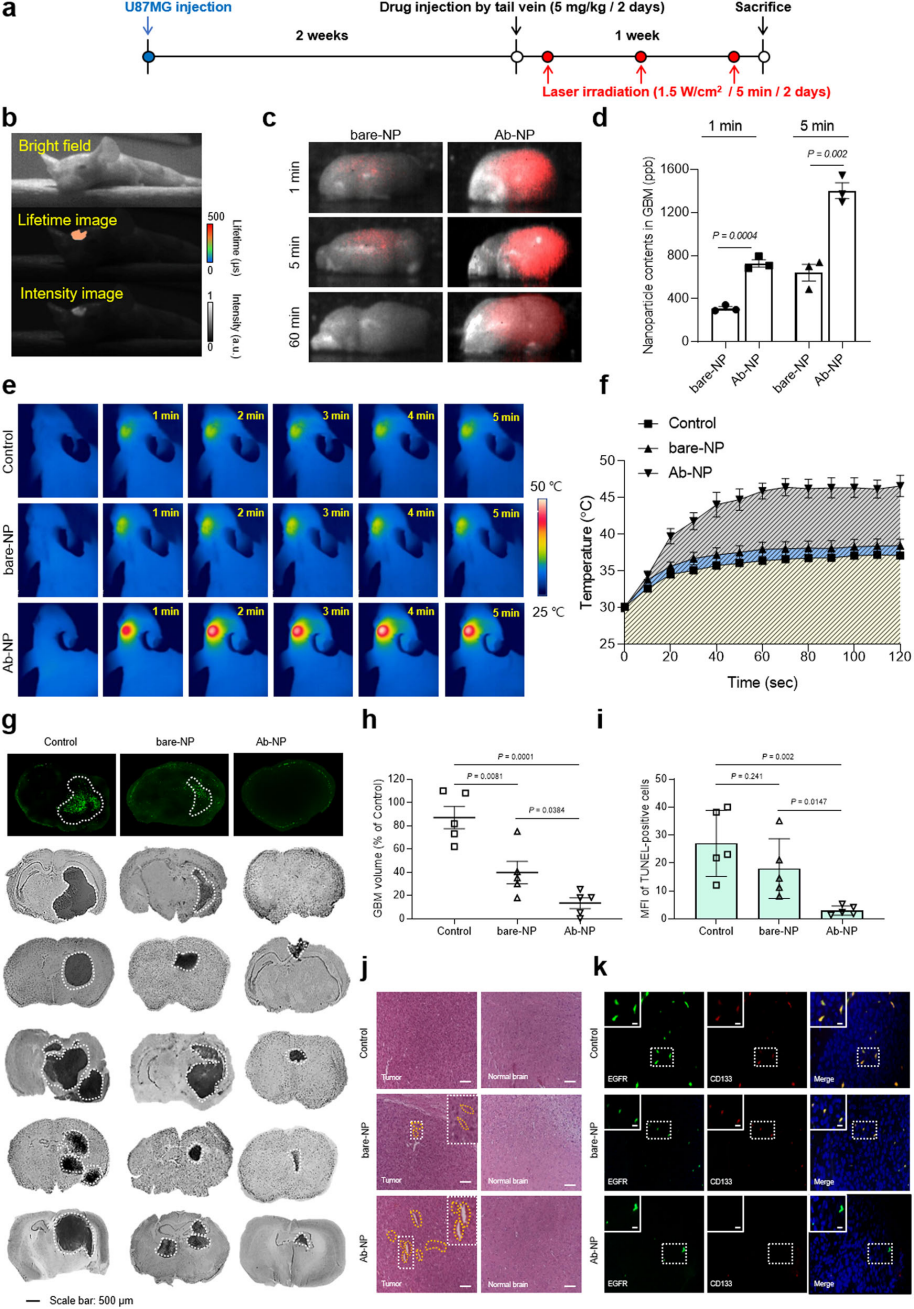
Lifetime imaging and PTT effects on the GBM-bearing orthotopic mice model. **a** Schedule of GBM orthotopic mice modeling and treatments. The drug was administered at a concentration of 5 mg/kg, and the laser intensity was set at 1.5 W/cm^2^. Treatments were conducted three times a week. **b** Lifetime and NIR images of mice brain 5 min after Ab-NP tail vein injection. **c** Comparison of GBM targeting of bare- and Ab-NPs via ex vivo lifetime images of mice brains 1, 5, and 60 min after tail vein injection. **d** Quantification of bare- and Ab-NPs accumulated in GBM after tail vein injection. Data are expressed as mean ± SEM (*n* = 3 biological independent animals). Two-sided P values calculated by Student’s *t* tests. **e** Thermal images of GBM lesions by laser irradiation for 5 min in a mice model in which PBS-vehicle, bare- and Ab-NPs were administered by tail vein injection. **f** Temperature elevation of the GBM lesion irradiated with a laser for 120 s. Data are expressed as mean ± SEM (*n* = 3 biological independent animals). **g** Representative images from the TUNEL assay and five individual brain sections with Nissl staining. The white dashed lines indicate the tumor regions. **h** Quantification of tumor volume after PTT with PBS-vehicle (control), bare- and Ab-NPs. Data are expressed as mean ± SEM (*n* = 5 biological independent animals). Two-sided *P* values calculated by Student’s *t* tests. **i** Quantification of TUNEL MFI. Data are expressed as mean ± SEM (*n* = 5 biological independent animals). Two-sided *P* values calculated by Student’s *t* tests. **j** H&E staining of brain section. Yellow dashed lines indicate thrombosis and hemorrhage. The results were representative of three independent experiments. Scale bars: 50 μm. **k** Immunofluorescence staining results for anti-EGFR antibody (green), anti-CD133 antibody (red), and DAPI (blue) in GBM-bearing brain sections. The results were representative of three independent experiments. Scale bars: 5 μm. Source data are provided as a Source Data file.

To investigate the NIR long-lifetime imaging and PTT effect of Ab-NPs in a GBM animal model, we injected U87MG into a BALB/c nude mouse and constructed an orthotopic GBM model. Mice were treated with PBS-vehicle (control), bare-NPs, and Ab-NPs via tail vein injection after two weeks of GBM modeling. After 5 min of drug administration, the PTT laser was irradiated to the GBM lesion for an additional 5 min. These treatment cycles were repeated 3 times over 1 week (Fig. 6a). During the first treatment, we monitored lifetime imaging on the GBM lesion with the 980 nm pulsed laser and it was monitored in NIR long-lifetime image than that of NIR intensity-based imaging method (Fig. 6b). Furthermore, the accumulation and clearance profiles of administered- bare-NPs and Ab-NPs in GBM tissues were monitored for 60 min (Fig. 6c and Supplementary Fig. S15). As a result, the NIR luminescence signal from Ab-NPs in GBM tissue was maximized at 5 min (mean intensity of 27.6 a.u.) and maintained until 60 min (mean intensity of 9.8 a.u.). However, bare-NPs were monitored for the same period (mean intensity of 5.0 a.u. at 5 min) and no signal was detected at 60 min. This could be interpreted as being due to the sufficient accumulation of Ab-NPs in the GBM tissue. The amounts of intravenously injected NPs in GBM, quantified by inductively coupled plasma-mass spectroscopy (ICP-MS), were 2.4- and 1.9-fold higher in Ab-NPs compared with bare-NPs at 1 and 5 min, respectively (Fig. 6d). Enhanced permeability and retention (EPR) are common events in GBM which caused by abnormal angiogenesis and tumor pressure. The accumulation of bare-NPs in the GBM tissues (~707.9 ppb, after 5 min intravenous injection) is therefore expected to be the result of the EPR effect, which is caused by the combination of increased extravasation (enhanced permeability) and a decreased drainage by the lymphoid system (increased retention). However, a more pronounced accumulation of Ab-NPs (~1370.0 ppb, after 5 min intravenous injection) was attributed to their interaction with CD133 expressed in GBM.

The temperature of GBM lesions under laser irradiation was approximately 10 °C higher than that of bare-NPs treatment (Fig. 6e, f) owing to the enhanced GBM-targeting of Ab-NPs. Because the Ab-NP treatment was repeated three times, the tumor volume was reduced by 59.4 ± 3.6 and 78.9 ± 4.5% with respect to those in the bare-NP-treated and control groups, respectively. Most tumor masses were significantly cleared in the five individual mice treated with Ab-NPs (Fig. 6g, h). TUNEL assays are widely used to identify apoptotic events by labeling the blunt ends of double-stranded DNA fragments with terminal deoxynucleotidyl transferase (TdT). Therefore, a TUNEL-positive signal should not be observed in the GBM area as the anti-apoptotic pathway in tumors is abnormally elevated, allowing cancer cells to resist apoptosis. However, significant flaws in the TUNEL assay’s interpretation of apoptosis have been identified during the last decade^34^. According to several studies, TUNEL staining is non-specific in the sense that it identifies any free 3′-hydroxyl ends, regardless of the chemical mechanism developed as expected. On the contrary, it also stains non-apoptotic cells such as necrotic degenerative cells^35^. Therefore, recent studies suggest that the morphology should be examined concurrently with the TUNEL assay to discriminate between apoptotic and necrotic cells. Consistent with recent claims, TUNEL-positive cells were predominated in the innermost GBM area where a large area of necrotic tumor cells was observed in Nissl staining (Fig. 6g). In this respect, TUNEL-positive cells might be necrotic tumor cells that contribute to the growth and aggressiveness of GBM. As a result, the mean fluorescence intensity (MFI) of the control group was measured to be 29.4 ± 11.6, whereas the Ab-NP treatment decreased to 3.2 ± 2.1 (Fig. 6i).

Furthermore, there was no apparent damage to the normal brain tissues which surround the GBM area, rather robust PTT-induced responses were observed at the tumor site such as hemorrhage and thrombosis (Fig. 6j). These results demonstrated that the 1.5 W/cm^2^ intensity of laser sufficiently facilitated PTT while avoiding laser-induced side effects on normal brain tissues. However, to evaluate the effect of the 808 nm laser, it is necessary to further optimize the intensity and irradiation time of the laser to precisely modulate the photothermal effect.

Epidermal growth factor receptor (EGFR) is associated with a malignancy phenotype that is distinct from the low levels of brain tumors and has been found in more than 50% of GBMs^36^. Additionally, the CD133 and a cancer stem cell marker co-localized with EGFR, increased phosphorylation, and stability of EGFR, acquiring stem cell-like properties that progressed to more malignant GBM^37,38^. In this regard, the fluorescence signals of EGFR and CD133 in GBM tissue were investigated to identify the severity of tumor progression and CD133 targeting efficacy, respectively. The fluorescence signals of EGFR and CD133 were most abundant in the control group and those signals were mostly co-localized in the merged image (Fig. 6k, results in the control group). Therefore, the correlation between EGFR and CD133 in the progression of GBM to malignancy was demonstrated. In the bare-NP-treated group, the distribution rate of EGFR was slightly decreased compared to the control group, but the fluorescence signal of CD133 and colocalization between EGFR and CD133 were continuously detected (Fig. 6k, results in the bare-NPs group). Interestingly, the fluorescence signal of CD133 and colocalization were no longer detected in the Ab-NP-treated group, which can be evidence of the Ab-NPs targeting CD133 which is expressed in GBM as intended. This is because once CD133 is engaged by its ligand, endocytosis occurs and is no longer expressed on the outer membrane, which can be stained by the antibody. Moreover, the fluorescence signal of EGFR was reduced compared to other treatment groups (Fig. 6k, results in the Ab-NPs group). Therefore, these results indicate that the GBM treatment strategy of PTT using Ab-NPs was successfully achieved.

### Biodistribution and toxicity study of intravenously injected bare-NPs and Ab-NPs

The biodistribution of bare-NPs and Ab-NPs was investigated via NIR luminescence imaging for 60 min. In general, the gap between vascular endothelial cells is less than 2 nm, which limits the excretion of NPs from the blood circulation after intravenous injection^39^. In this study, bare-NPs and Ab-NPs have a diameter of approximately 40–50 nm, therefore they were expected to pass through fenestrae with a diameter of up to 200 nm in the endothelium of the liver, kidney, and lung, whereas other tissues have a diameter less than 6 nm or no fenestrae^40^. As expected, both bare-NPs and Ab-NPs are continuously excreted from the reticuloendothelial system (RES) such as the liver, kidney, and lung until 60 min (Supplementary Fig. S15).

The major limitations for the in-development of nanomedicines are their unclear elimination profile in the RES organs and toxicity. Thus, we performed histopathological analyses of the major organs (heart, lung, liver, kidney, spleen, and intestine) after intravenous administration of three repeated doses at a concentration of 5 mg kg^−1^ in the mouse model. As a result, there was no detection of immune responses leading to histological lesions composed of inflammatory cells such as macrophages, neutrophils, and B lymphocytes in both bare-NP and Ab-NP-treated groups (Supplementary Fig. S16). Weight loss, an important parameter in repeated-dose toxicology studies, was also not identified throughout the treatment cycles (Supplementary Fig. S17). After treatments are terminated, the spleen weight/body weight ratio was evaluated to investigate whether spleen enlargement occurred due to unwanted immune stimulation after repeated drug doses. As expected, there was no significant difference compared to the control (Supplementary Fig. S18).

Next, biochemical, and complete blood count (CBC) tests, a series of blood tests to evaluate the functionality of vital organs and systems, were conducted by drawing blood after three repeated doses of either bare- or Ab-NPs for one week (Supplementary Fig. S19). No side effects such as inflammation, infection, or anemia occurred after repeated doses of NPs, and the functionality of organs was retained within the normal range. It can be collectively inferred that repeated intravenous injection of bare-NPs or Ab-NPs does not cause an unnecessary immune response and has no systemic toxicity. However, a concrete investigation of long-term toxicity must be performed in future studies.

## Discussion

In terms of enhancing the PTT effect, herein, we have introduced a novel approach to directly heat water molecules, the main components of the body (60–65%), using a certain luminescence wavelength of lanthanide-doped nanomaterials. The 1.0 μm emission wavelength from water-heating NPs is strongly absorbed by water molecules since their second overtone of the stretching band gives a significant absorption ability around 950–1050 nm region^41,42^. However, in the cyclohexane solution, there was no absorption around 950–1050 nm (Fig. 2c, d). This observation, therefore, indicates that the light energy of 1.0 μm can be converted to thermal energy by the strong absorption derived from the overtones and the stretching vibrations of O–H oscillators in water molecules^43^. As a result, developed water-heating NPs have shown great potential for photothermal materials with a photothermal conversion efficiency of 23.3% in an aqueous solution. This remarkable photothermal performance of water-heating NPs in the aqueous solution indicates that the PTT efficacy reached the lethal temperature (>50 °C), making it a sufficient photothermal agent in a water-rich biological environment (Fig. 2d). Comparatively, only CR-induced photothermal conversion efficiency was found to be 8.8% which is consistent with previously reported only Nd-doped nanomaterials^8,10^. In addition, homogeneously structured water-heating NIR NPs showed bright NIR luminescence with a lifetime on the order of microseconds (423 ± 6.5 µs). This micro-scale lifetime can be easily distinguished by the time-gating technology from autofluorescence, which is considered a nano-scale lifetime such as NADH, collagen, tryptophan, and melanin^44^. Figure 4e, f clearly verifies the luminescence against the background signal, and thus the lifetime imaging technology was very useful and suitable for deep-tissue imaging without a thermal increase (>3 °C).

For a clinical approach, we additionally conjugated the GBM-targeting ligand, anti-CD133 mAb, to water-heating NIR NPs (Ab-NPs). CD133 is a transmembrane protein preferentially expressed in the membranes of GBM cells, and the interaction between anti-CD133 mAb and CD133 can induce endocytosis, thereby an ideal target for the delivery of water-heating NIR NPs to improve GBM therapy^45^. Moreover, CD133 was reported to be a necessary factor for GBM cells to adapt to conventional therapies (radio- and chemotherapy), migrate between different clonal populations of tumors, and re-emerge to initiate tumor relapse. In recent clinical and translational studies, targeting CD133 with an anti-CD133 antibody significantly improved the efficiency of immunotherapy in patient-derived GBM models^46^. Therefore, we aimed to utilize an anti-CD133 antibody to overcome the resistance of conventional therapies and successfully target and treat GBM. NIR long-lifetime imaging, PTT, tumor targeting, biodistribution, and side effects of systemic administered Ab-NPs were investigated in an orthotopic GBM-bearing animal model. As a result, the GBM-targeted Ab-NPs induced intracranial NIR long-lifetime imaging with a high-resolution under 980 nm pulsed laser irradiation. In contrast, under the 808 nm CW laser irradiation, the local water-molecular heating effect by the GBM-targeted Ab-NPs showed satisfactory PTT performance on GBM (78.9% tumor volume reduction). In general, the tumor tissues have a denser structure and consist of polymorphic cells with a diameter of 10–20 μm and elliptical nuclei, which makes it possible to distinguish the tumor region from brain tissues. Because of PTT, hemorrhage and thrombosis at the tumor site were mostly observed in the Ab-NPs treated group, which was absent in the control group (Fig. 6j). This demonstrates that the PTT using Ab-NPs irreversibly damages endothelial cells and vascular membrane. Since tumor growth is closely related to vasculature due to the oxygen and nutrient supply, microvasculature destruction by PTT damages tumor blood vessels, resulting in hemorrhage and destroying the tumor^47^. To corroborate the robust response of PTT with another methodology, the expression level of CD133 and EGFR were investigated in the PTT-applied tumor region. Recently, the cancer stem cells in GBM have received great attention due to their high oncogenic properties and prominent roles in mediating tumor re-growth, self-renewal, and therapeutic resistance^48^. Among molecules used in combination or individually to identify glioma stem cells, CD133 is one of the most important stem cell surface markers associated with higher invasiveness and worse prognosis^46^. Typically, CD133-positive tumor cells represent more detrimental resistance to surgical resection compared to CD133-negative cells and augmented CD133 expression is observed in residual cancer cells after adjuvant therapy^49^. Moreover, the molecular mechanism of CD133 is highly involved in the expression of EGFR, which is overexpressed in approximately 50% of conventional GBM. In this regard, CD133 serves to sustain aberrant EGFR-AKT signaling which is a pivotal mechanism affecting manifest resistance to treatment. Based on the above, we examined the expression level of both CD133 and EGFR after PTT with Ab-NPs, which in turn decreased, and further demonstrated that the treatment strategy of water-heating NIR NP-mediated PTT could be rational and feasible. Meanwhile, side effects derived from the biodistribution of NPs were not found during repeated systemic administration.

In this study, we anticipate that the present multifunctional water-heating NIR NPs will be attractive nanomaterials to enhance the overall effectiveness of brain tumor therapy with NIR long-lifetime imaging-guided PTT.

## Methods

### Experimental cells lines and animals

Human glioblastoma cell line (U87MG; Korean Cell Line Bank, Seoul, Korea) derived from human brain cancer. U87MG cells were cultured using Dulbecco’s Modified Eagle’s Medium (DMEM, high glucose, Welgene, Gyeongsangbuk-do, Korea) containing 10% Fetal Bovine Serum (CELLectTM, Gold, 13 US Origin), 1% penicillin-streptomycin (Gibco, USA) in standard culture conditions at 37 °C 5% CO_2_. All animals were housed in specific pathogen-free conditions and maintained under the Institutional Animal Care and Use Committee (IACUC: 2020–0081) at Hanyang University. The animal ethics guidelines of the Institutional Animal Care and Use Committee (IACUC) at Hanyang University were strictly followed, ensuring that the maximum volume of the mouse tumor did not exceed 1 cm^3^.

### Reagents

Neodymium(III) acetate hydrate (99.9%), yttrium(III) acetate hydrate (99.9%), ytterbium(III) acetate hydrate (99.9%), thulium(III) acetate hydrate (99.95%), oleic acid (OA, 90%), 1-octadecene (ODE, 90%), sodium hydroxide (NaOH, ≥98%), ammonium fluoride (NH_4_F, ≥99.9%), ethanol (absolute), methanol (≥99.8%), cyclohexane (≥99%), tetrahydrofuran (THF, ≥99.9%), dopamine hydrochloride (≥99.9%), hydrochloric acid (HCl, 37%), dimethyl sulfoxide (DMSO, ≥99.9%), hydroxylamine hydrochloride (HH, 99%), N,N-dimethylformamide anhydrous (DMF, ≥99.8%), nitrosyl tetrafluoroborate (NOBF_4_ ≥ 95%), and PAA solution (*M*_*w*_ = 2000 g/mol) were purchased from Sigma-Aldrich, St. Louis, MO, USA. HEPES buffer (1 M) was purchased from Gibco. *N*-succinimidyl S-acetylthioacetate (SATA), sulfosuccinimidyl 4-(N-maleimidomethyl) cyclohexane-1-carboxylate (sulfo-SMCC), and CD133 (Prominin-1) monoclonal antibody (12-1338-42) was purchased from Thermo Fisher Scientific, Waltham, MA, USA. An Amicon Ultra centrifugal filter (0.5 mL, 30 K, 5 K) was purchased from Millipore (Bedford, MA, USA). Anti-EGFR antibody (ab40815), goat anti-rabbit IgG-H&L Alexa Fluor 488 (ab150077), and goat anti-rabbit IgG H&L Alexa Fluor 647 (ab150079) were purchased from Abcam, UK. A DAPI mounting kit was purchased from Vector Laboratories, Inc., Burlingame, CA, USA.

### Synthesis of NPs

Lanthanide-doped NPs were synthesized by the thermal decomposition of lanthanide acetate precursors. In a typical synthesis process, Ln(CH_3_CO_2_)_3_ (Ln = Y, Nd, or Yb total 0.4 mм) was mixed with oleic acid (3 mL) and 1-octadecene (7 mL). The mixed solution was then heated to 150 °C, held for 30 min, and cooled to 50 °C. Next, a methanolic solution of NaOH (1 mм) and NH_4_F (1.6 mм, 5 mL) was continuously added to the oleate–lanthanide mixture solution and stirred at 50 °C. The obtained solution was then heated to 100 °C and degassed through the vacuum pump to remove the residual water and methanol. Thereafter the resultant solution was heated to 300 °C, maintained under argon for 1 h, and cooled to room temperature. The precipitated NPs were collected by centrifugation and washed. The final product was re-dispersed in cyclohexane (2 mL).

### Surface modification

#### Dopamine-modified NPs

NPs (15 mg) were dissolved in THF, while dopamine hydrochloride (50 mg) was dissolved in water. The solutions were mixed in a flask (50 mL) and heated to 50 °C with vigorous stirring. Following the HCl addition, the resultant NH_2_-ligand was collected through several washing steps. To exchange maleimide ligand-modified NP, NH_2_-NP (2 mg) was dispersed in HEPES buffer (10 mм, 200 µL). sulfo-SMCC was dispersed in HEPES buffer (10 mм) and then, these solutions were mixed and incubated for 5 h. To prepare antibody-conjugated NP, SATA stock solution was added to the pristine antibody (50 µg) for 30 min. HH solution (1.75 µL, 0.5 м) was continuously added to the above solution and incubated for 2 h. The resultant solution was washed using a 30-k centrifuge filter. Subsequently, thiol-modified antibodies and maleimide-modified NPs were linked by a click reaction. The antibody concentrations were determined by the absorption spectrum at 280 nm. Finally, the surface of the antibody-NPs was covered with a 1% BSA solution as a blocking agent to minimize non-specific binding.

#### PAA-coated NPs

NPs (15 mg) were suspended in DMF, while NOBF_4_ (25 mg) was dispersed and mixed vigorously. The reaction was incubated under shaking at 160 g for 60 min. NOBF_4_-coated NPs were washed with 4 mL of chloroform and centrifuged twice at 11,000 *g* for 5 min.

After the NOBF_4_ ligand exchange, the NPs were coated with PAA by adding a 10 % PAA solution (pH 9). The reaction was incubated for 24 h at 60 °C. The final PAA-coated NPs were washed twice with 1 mL of water by centrifugation at 11,000 *g* for 15 min.

#### Ligand-free NPs

After coating the NPs with NOBF_4_, they were incubated for 2 h in 0.2 M HCl at RT. The final ligand-free NPs were washed twice with 1 mL of water by centrifugation at 11,000 *g* for 15 min.

#### Characterization of water-heating NIR NP

Sample images were analyzed on a JEN-2100F (JEOL. Ltd.) installed at the Hanyang LINC3.0 Center for Research Facilities (Seoul, Republic of Korea). The XRD patterns of the 1.0 µm NPs were recorded by an XRD-7000 diffractometer. A Zetasizer Nano ZSP instrument (Malvern Co., UK) was used to determine the zeta potentials of the water-heating NIR NPs. The Fourier transform infrared (FT-IR) spectra of the water-heating NIR NP were obtained by using an iS10 Fourier transform infrared spectrophotometer (Thermo Fisher Scientific Co., USA). The upconversion photoluminescence emission spectra were recorded using a Flame spectrometer (Ocean Optics, Inc., USA) under external excitation at 980 nm provided by an infrared diode laser (Changchun New Industries Optoelectronics Tech. CO., China). A Jasco V-700 UV–Vis spectrophotometer (Jasco., Japan) was used to record the absorbance spectra. The lifetime and images were obtained by the iStar intensified sCMOS camera (Andor) using time-gated imaging technology. Their time gate delay was 100 μs, and the time gate width was 50 μs. Sample images and lifetime images were analyzed by using Andor software (Solis 64) and ImageJ program (FlimJ-plugin). All images were obtained under the irradiation of a 980 nm pulsed laser (CNI, MDL-III-980-2W) with a power density of 0.5 W m^−2^. The NIR emission wavelength was selectively measured using a bandpass filter (Semrock, ff-01-800/12-25, Edmund 980 nm CWL, 12.5 mm Dia., Hard Coated OD 4.0 10 nm) and shortpass filter (Semrock, ff-01-950/sp-25) placed in front of an all detectors.

#### Photothermal efficiency

The photothermal conversion efficiency of water-heating NIR NP was calculated by the equation described by Roper et al. (Supplementary Fig. S7).

1 $$\eta=\frac{{hS}\left({T}_{\max }-{T}_{{surr}}\right)-{Q}_{{{{{{{\rm{Dis}}}}}}}}}{I(1-{10}^{-{A}_{808}})}$$

where η is photothermal conversion efficiency, *h* is a heat transfer coefficient, *S* is the surface area of the container, Δ*T* represents the temperature difference of the surroundings, *I* is the power density of the laser, *A*_808_ is the absorbance intensity of NPs in water solution at 808 nm, and the *Q*_*Dis*_ represents the heat induced by solvent absorption. *hS* can be evaluated by the equation below:

2 $${hS}=\frac{m{C}_{p}}{{\tau }_{s}}$$

In our experiments, *m* is the mass of the solution (0.1 g), *C*_*p*_ is the heat capacity of the solution (4.2 J g^−1^), and τ_s_ is defined by the time data versus −lnθ (85.14 s, Supplementary Fig. S7b). Δ*T* is 30.8 °C (ca. 50.8–20), and *Q*_*Dis*_ is the heat dissipated from the light absorbed by the quartz sample cell, which was independently measured to be 25.1 mW using a quartz cuvette cell containing distilled water. *I* was 2.0 W cm^−2^, *A*_808_ was 0.15, and using these values, the photothermal conversion efficiency was found to be 23.3%.

#### Preparation of orthotopic GBM-bearing mouse model

To prepare the orthotopic GBM-bearing mouse model, GBM cells (U87MG cell) were intracranially injected into 7-week-old male Balb/c nude mice (*n* = 15) that purchased from. Nara-Bio Company, Seoul, Korea. Briefly, male nude mice were anesthetized with isoflurane (3%) and fixed by ear bar in a stereotaxic instrument (Stoelting Co., IL, USA). Once each mouse was anesthetized, the scalp at the surgical position was removed, and a small hole positioned at 2 mm right lateral and 2 mm posterior from the bregma was drilled under sterile conditions. PBS (containing 1 × 10^6^ U87MG cells, 8 μL) was loaded into a 26-G Hamilton syringe (Hamilton Company, NV, USA), and then the syringe was placed on the stereotaxic apparatus. After the needle of the syringe was positioned at a 3 mm depth, cells were injected with a 1 μL min^−1^ injection rate, followed by 3 min of waiting time to prevent overflow. After injection, the hole was sealed with bone wax and the scalp was closed with suturing. After this procedure, the mice were kept for 3 weeks until the injected cells reached the appropriate size of GBM tissue. 2 weeks of post-surgery, in vivo experiments were undertaken.

#### Cell culture and MTT assay

U87MG cells were cultured on a T-75 cell culture plate with DMEM (10 mL) containing 10% FBS and 1% penicillin-streptomycin in a cell incubator under environmental conditions of 5% CO_2_ and 37 °C. When the cells attained more than 70% confluency, they were washed twice with DPBS, treated with Trypsin-EDTA (3 mL), and reacted at 37 °C at a constant humidity to separate the cells. After collecting the cells by centrifugation, cells (10 µL) and trypan blue (10 µL) were mixed, the cells were counted using a hematocytometer, and were diluted with a culture medium to the required concentration for each experiment. The cytotoxicity of NPs was evaluated by calculating the cell viability by MTT assay. U87MG cells were seeded in 96-well plates at a density of 1 × 10^4^ cells per well for 24 h. Cells were treated with UCNP-CD133 at various concentrations of 0, 25, 50, 100, 200, and 400 µg mL^−1^, respectively. After reacting for 24 and 72 h in a dark room without light, MTT reagent (100 µL, 0.5 mg mL^−1^) was added to each well, and the cells were further cultured for 4 h. Then, the excess MTT solution was removed from each well and the formed formazan was dissolved in DMSO (100 µL). The absorbance of the MTT product was acquired at 570 nm using a Varioskan Flash spectral scanning multimode reader (Thermo Fisher Scientific Inc., USA). Cell viability for each concentration was assessed in triplicate and determined relative to untreated control cells. The following formula was used to calculate the viability of cell growth: Cell viability (%) = (absorbance value of treatment group – blank)/(absorbance value of control group – blank) x 100%

We investigated the concentration- and irradiation dose-dependent PTT effect of Ab-NP on cell viability. U87MG cells were incubated with various concentrations of Ab-NP (0, 50, 100, 200, 400, and 800 μg mL^−1^) in 96-well plates, and cells were washed 3 times with PBS to remove excess NPs. Next, the cells were exposed to 808 nm irradiation respectively for 0, 10, 20, and 30 min. Controls were used as water-heating NIR NP with the same loading concentration and duration of NIR exposure. Finally, cell viability was measured by MTT assay after an additional 24 h incubation in the dark.

#### Flow cytometry analysis

To monitor the effects of cytotoxicity, an analysis was performed using a flow cytometer (FACS Canto, BD Biosciences). U87MG cells were incubated at 37 °C for 24 h in 24-well microplates and treated with Ab-NP (200 µg ml^−1^). After removing the excess NPs, the cells were subjected to NIR irradiation for 0, 10, and 20 min, and then Sytox-Green was added. Before FACS analysis, cells cultured in microplates were detached from the wells using Trypsin-EDTA and centrifuged at 1000 *g* for 3 min. Cells were filtered through a 100 μm cell strainer and resuspended in 1 × DPBS (200 μL) containing 5% FBS. Between 10,000 and 20,000 events were collected using a forward scatter threshold of 10,000. Data were collected on pulse height, area, and width parameters for each channel. SG collected data using a 488 nm laser and a 530 nm bandpass filter. Flow cytometry data were then analyzed and plotted in FCS format in a histogram analysis using FlowJo software (ver. 10.8.1., FlowJo, LLC).

#### Histological study on the orthotopic GBM-bearing mouse model

Tissues were immobilized in paraformaldehyde (4%) for 2 days and then placed in a Leica TP1020 Semi-enclosed Benchtop Tissue Processor (Wetzlar, Germany) for washing, dehydration, clearing, and paraffin infiltration of the tissue samples, followed by embedding in paraffin blocks. The blocks were transversely sectioned at a thickness of 6 μm with a Leica RM2145 Microtome (Wetzlar, Germany). For Nissl staining, the brain slides were stained with a stain solution prepared by dissolving cresyl violet-acetate (0.2 g) in distilled water (150 mL) and a buffer solution containing acetic acid (0.1 м) and sodium acetate (0.1 м). H&E staining was conducted following the manufacturer’s instructions. For immunofluorescence staining, GBM tissue slides were stained with anti-EGFR antibody and anti-CD133 antibody and, diluted 1:100 in phosphate-buffered saline Tween-20 (PBST) and goat serum mixture. Next, goat anti-rabbit IgG-H&L Alexa Fluor 488 and goat anti-rabbit IgG H&L Alexa Fluor 647 were used as secondary antibodies, followed by DAPI mounting.

#### Biochemical and hematological tests

We procured 24 male BALB/c normal mice of seven-week-old from Nara-Bio Company, Seoul, Korea, for the experiment. Male BALB/c normal mice were intravenously administered with PBS, bare-NPs, and Ab-NPs three times over one week. Their blood was collected by cardiac puncture to obtain serum (100 μL, for the biochemistry test) and plasma (200 μL, for the CBC test). Samples were referred to Daegun Health Care Co. (DK Korea, Seoul, South Korea) for CBC (*n* = 4) and biochemical tests (*n* = 4).

### Reporting summary

Further information on research design is available in the Nature Portfolio Reporting Summary linked to this article.

## Supplementary information

Supplementary Information

Reporting Summary

## Data Availability

The authors declare that all data supporting the finding of this study are available within the article and its supplementary information files. The remaining data are available upon reasonable request from the corresponding author J.L. or within the Source Data file. Source data are provided with this paper.

## Source data

Source Data
