# Supplementary material for: A local water molecular-heating strategy for near-infrared long-lifetime imaging-guided photothermal therapy of glioblastoma
Source: Nat Commun. 2023 May 13;14:2755. doi: 10.1038/s41467-023-38451-3 (PMC10183012; doi:10.1038/s41467-023-38451-3)
Supplement: Supplementary file 1 — Supplementary Information [file 41467_2023_38451_MOESM1_ESM.docx]

**Supplementary Information**

**A Local Water Molecular-heating Strategy for Near-Infrared Long-lifetime Imaging-guided Photothermal Therapy of Glioblastoma**

*Dongkyu Kang^1^*, Hyung Shik Kim^2^*, Soohyun Han^3^, Yeonju Lee^4^, Young-Pil Kim^,3,4,5,6^, Dong Yun Lee^2,5,7,8^**, Joonseok Lee^1,6^***

^1^Department of Chemistry, Hanyang University, Seoul 04763, Republic of Korea

^2^Department of Bioengineering, College of Engineering, and BK FOUR Biopharmaceutical Innovation Leader for Education and Research Group, Hanyang University, Seoul 04763, Republic of Korea

^3^Department of HY-KIST Bio-Convergence, Hanyang University, Seoul 04763, Republic of Korea

^4^Department of Life Science, Hanyang University, Seoul 04763, Republic of Korea

^5^Institute of Nano Science and Technology (INST), Hanyang University, Seoul 04763, Republic of Korea

^6^Research Institute for Convergence of Basic Sciences, Hanyang University, Seoul 04763, Republic of Korea

^7^Institute for Bioengineering and Biopharmaceutical Research (IBBR), Hanyang University, Seoul 04763, Republic of Korea

^8^Elixir Pharmatech Inc., Seoul 07463, Republic of Korea

** Contributed equally to this study*

****Corresponding author**

*E-mail address:* [*joonseoklee@hanyang.ac.kr*](mailto:joonseoklee@hanyang.ac.kr)*, dongyunlee@hanyang.ac.kr*


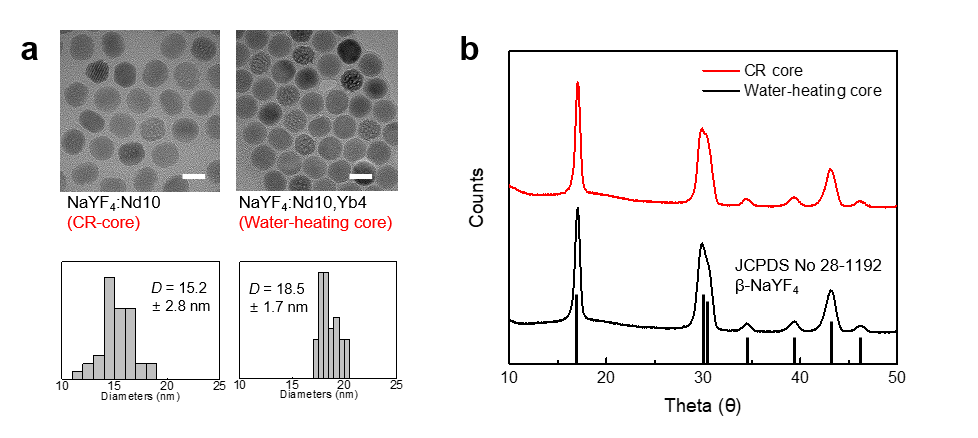


**Supplementary Figure S1.** Characterization of **water-heating cores**. a) TEM images and size distributions of **CR-** and **water-heating cores** (15.2 ± 2.8, and 18.5 ± 1.7, respectively), scale bars: 20 nm. 3-TEM images of each sample were included for statistical analysis, the results were presented as mean ± standard deviation. b) XRD patterns of core NPs. Source data are provided as a Source Data file.


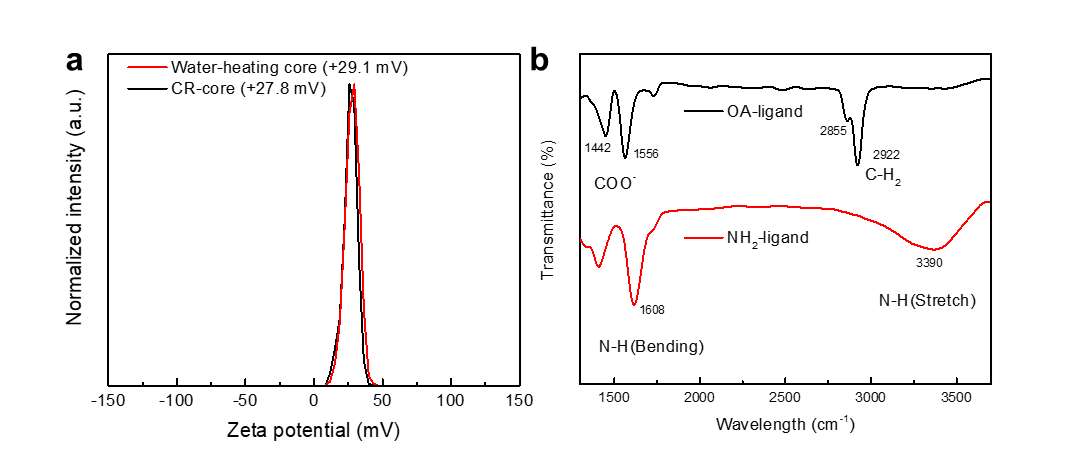


**Supplementary Figure S2.** a) Zeta potential profiles of dopamine-ligand exchanged **water-heating core** (+29.1 mV) and **CR-core** (+27.8 mV). The observed charges for each ligand demonstrated the homogeneously exchanged ligands of the NPs. b) FT-IR spectrum of the oleic acid ligand (black) and dopamine ligand (red). The surface modification was confirmed by the appearance of two new bands at 1608 and 3390 nm, attributed to N–H bending and stretching of amine groups, respectively. Source data are provided as a Source Data file.


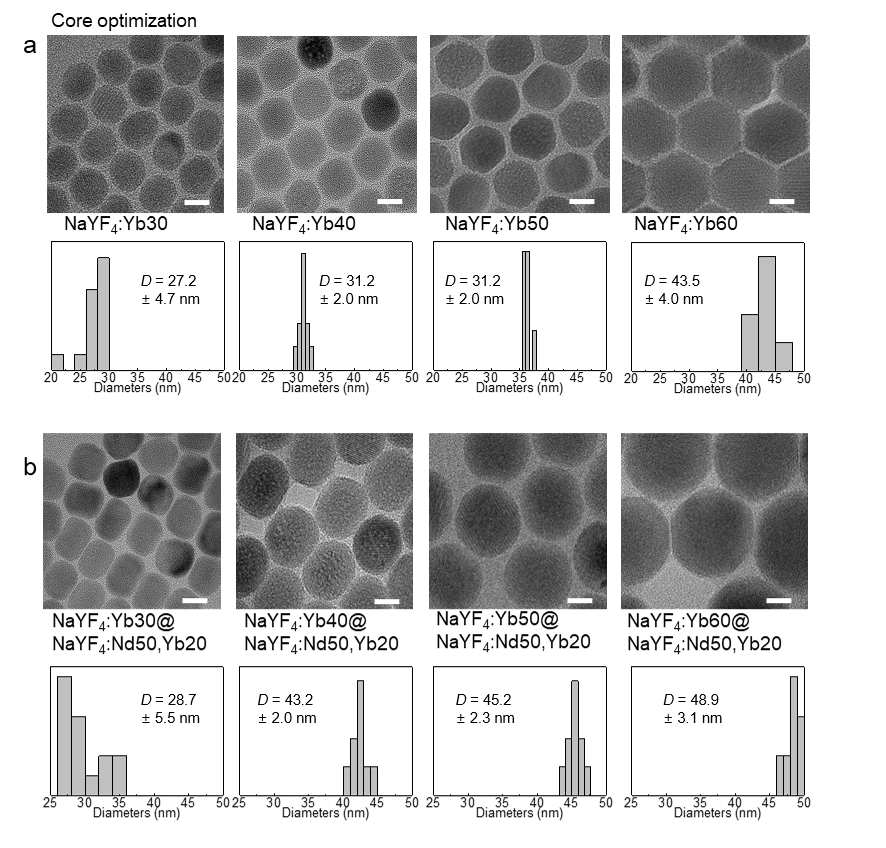


**Supplementary Figure S3.** Optimization of core NPs. TEM images and particle size distributions of a) core NPs with average diameters of (left to right) 27.2 ± 4.7, 31.2 ± 2.0, 36.4 ± 1.4, and 43.5 ± 4.0 nm and b) core@shell NPs with average diameters of (left to right) 28.7 nm ± 5.5, 43.2 ± 2.0, 45.2 ± 2.3, and 48.9 ± 3.1 nm, respectively. Scale bars: 20 nm. 3-TEM images of each sample were included for statistical analysis, the results were presented as mean ± standard deviation.

**
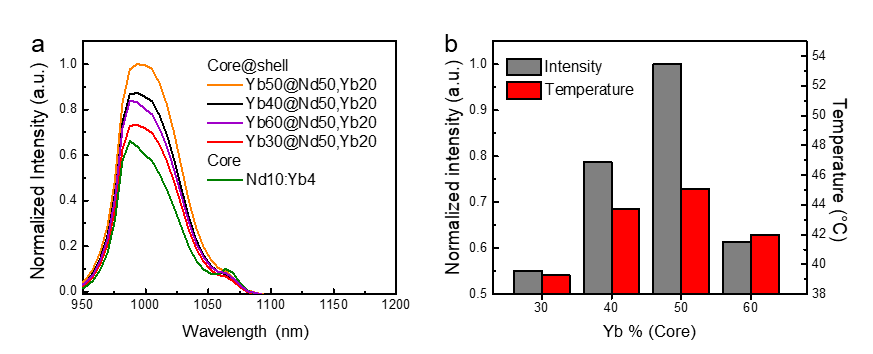
**

**Supplementary Figure S4.** Optimizing the Yb concentration of the core. a) 1.0 µm emission spectrum of core@shell NPs (NaYF_4_:x%Yb@NaYF_4_:20%Yb,50%Nd) with doped with various concentrations of Yb in the core and b) 1.0 µm emission intensities and the corresponding temperature profiles. Source data are provided as a Source Data file.


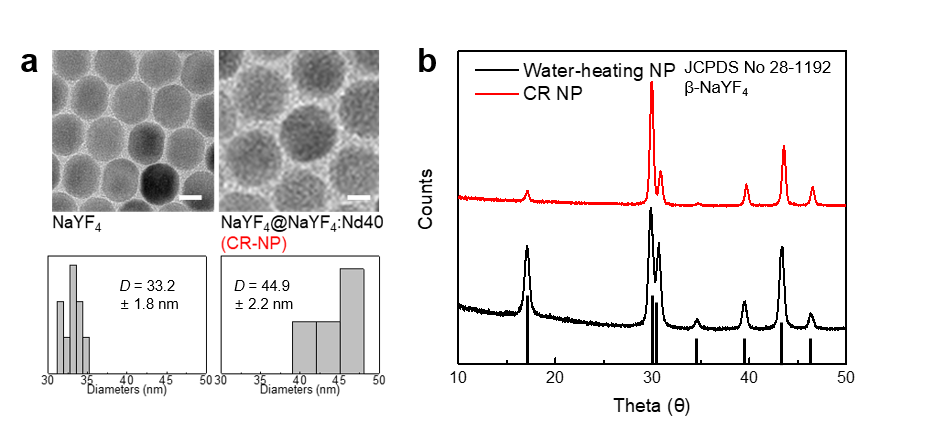


**Supplementary Figure S5.** a) TEM images and particle size distributions of CR NPs (core and core@shell NPs) with average diameters of 33.2 ± 1.8 nm and 44.9 ± 2.2 nm, respectively. Scale bars: 20 nm. 3-TEM images of each sample were included for statistical analysis, the results were presented as mean ± standard deviation. b) XRD patterns of CR NPs. Source data are provided as a Source Data file.

**Supplementary Figure S6**. Zeta potential profiles of **water-heating NIR NPs** conjugated with dopamine ligands (+36.5 and +36.4 mV for the **water-heating** and **CR NPs**, respectively), sulfo-SMCC ligands (-32.9 mV), and antibody ligands (-9.8 mV). Source data are provided as a Source Data file.

**
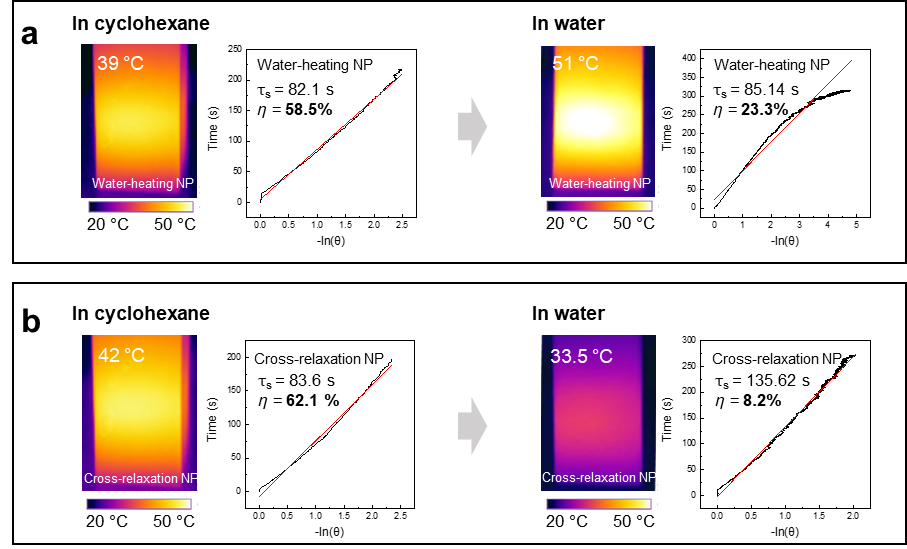
**

**Supplementary Figure S7**. a) Temperature profiles of **water-heating NPs** (NaYF_4_:50%Yb@NaYF_4_:40%Nd,20%Yb) (5 mg/mL) in cyclohexane and aqueous solution under the irradiation of a CW NIR laser (808 nm, 0.5 W/cm^2^ for cyclohexane and 2 W/cm^2^ for aqueous due to their different boiling points). Linear time data versus -ln(θ) obtained from the cooling period. b) Temperature profiles of **CR NPs** (NaYF_4_@NaYF_4_40%Nd) (5 mg/mL) in cyclohexane and aqueous solution under the irradiation of a CW NIR laser (808 nm, 0.5 W/cm^2,^ and 2 W/cm^2^). Linear time data versus -ln(θ) obtained from the cooling period. Detailed calculations are described in the methods section. Source data are provided as a Source Data file.


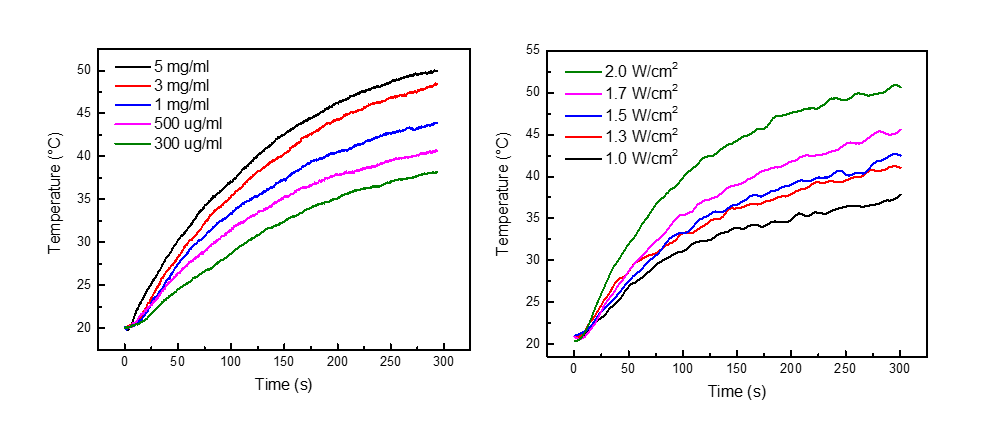


**Supplementary Figure S8.** Temperature profiles of water-heating NPs under the 808 nm laser with a) different concentrations (0.3, 0.5, 1, 3, and 5 mg/mL) at a power density of 2.0 W/cm^2^ for 5 min and b) different power densities (1.0, 1.3, 1.5, 1.7 and 2.0 W/cm^2^) at a concentration of 5 mg/mL for 5 min. Source data are provided as a Source Data file.


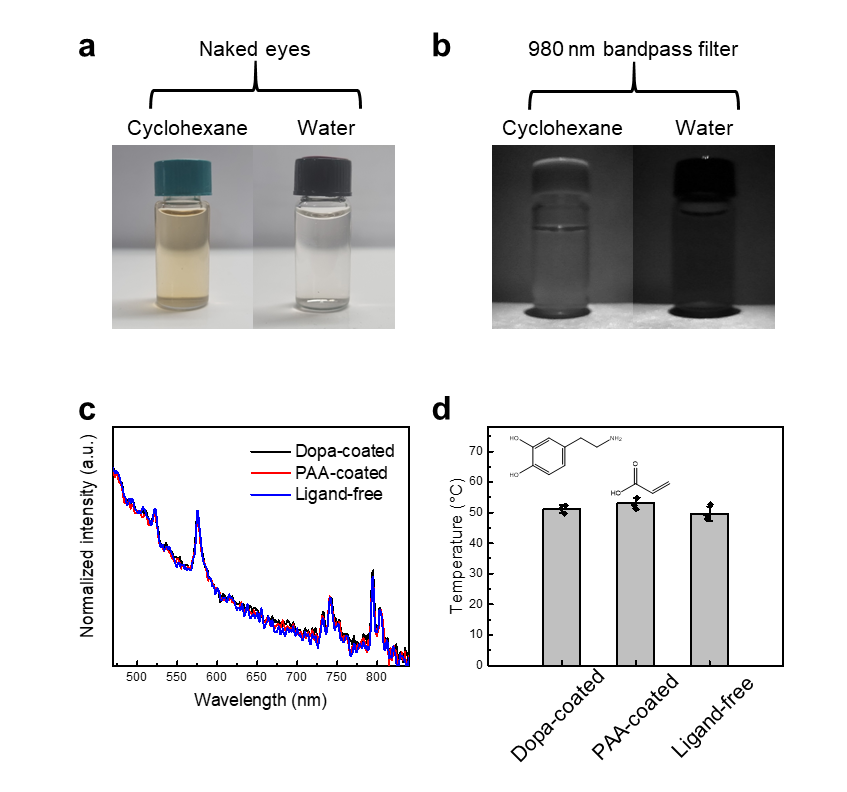


**Supplementary Figure S9.** Characterization of the water-heating NPs. a) Optical images of water-heating NPs in cyclohexane and water. b) NIR images of water-heating NP in cyclohexane and water with a 980 nm bandpass filter. c) Absorption spectrum and d) temperature profiles of dopamine-coated, PAA-coated, and ligand-free water-heating NPs. Data are expressed as mean ± SEM (n = 3, independent experiments) Source data are provided as a Source Data file.


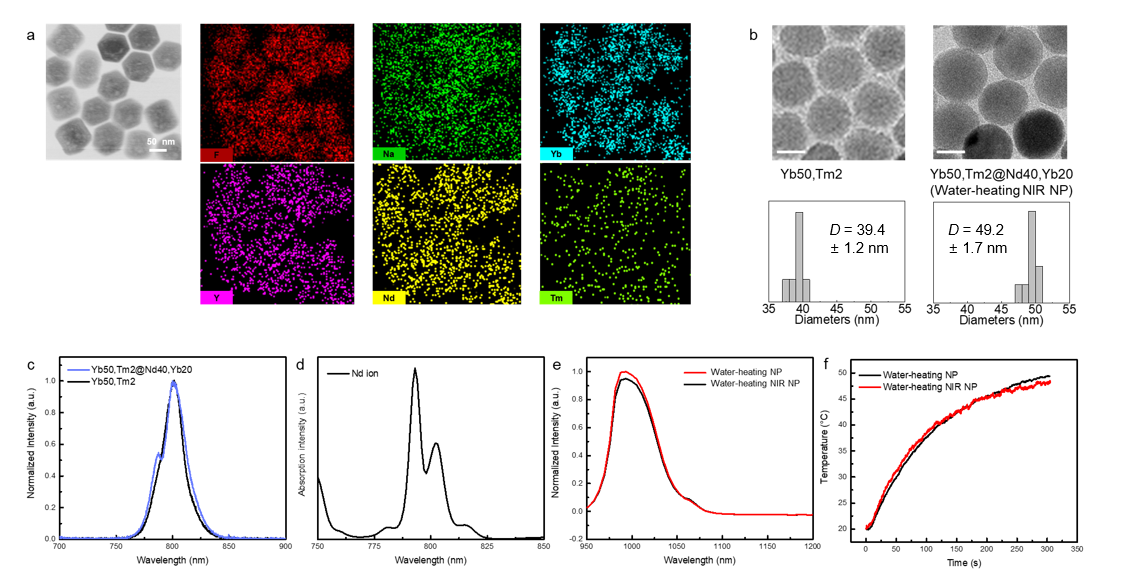


**Supplementary Figure S10.** Optical properties of **water-heating NIR NPs**. a) EDS mapping images of **water-heating NIR NPs**. b) TEM images of NaYF_4_:50%Yb,2%Tm and NaYF_4_:50%Yb,2%Tm@NaYF_4_:40%Nd,20%Yb (**water-heating NIR NPs**), (39.4 ± 1.2 nm and 49.2 ± 1.7 nm, respectively), scale bars: 20 nm. 3-TEM images of each sample were included for statistical analysis, the results were presented as mean ± standard deviation. c) NIR emission spectrum for 800 nm emission under 980 nm laser excitation. The 800 nm emission is slightly absorbed by the outer shell doped with Nd^3+^ ions. d) NIR absorption spectrum of Nd^3+^ ions e) 1.0 μm emission spectrum and f) time profile of **water-heating NIR NPs** and **water-heating NPs** in cyclohexane under 808 nm laser excitation. Source data are provided as a Source Data file.


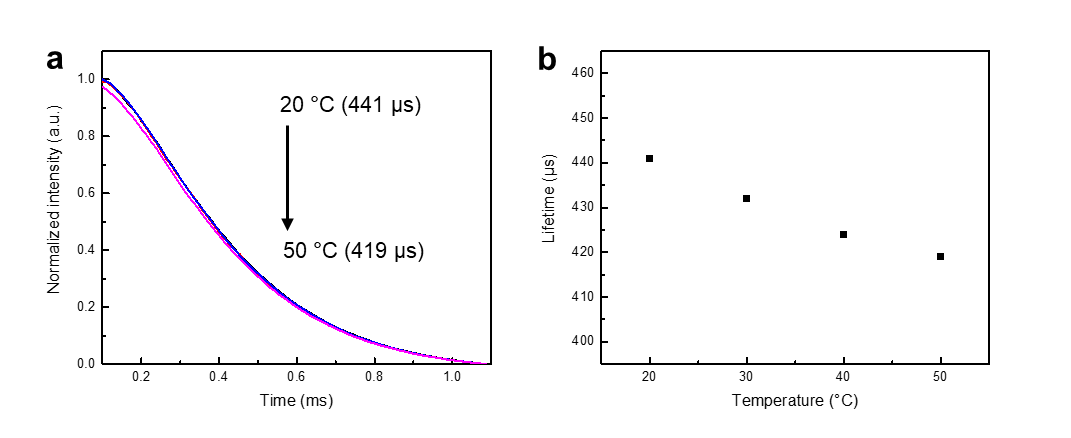


**Supplementary Figure S11**. Temperature-dependent lifetime of water-heating NIR NPs. a) Lifetime profiles and b) corresponding plots of the lifetime as a function of temperature. Source data are provided as a Source Data file.


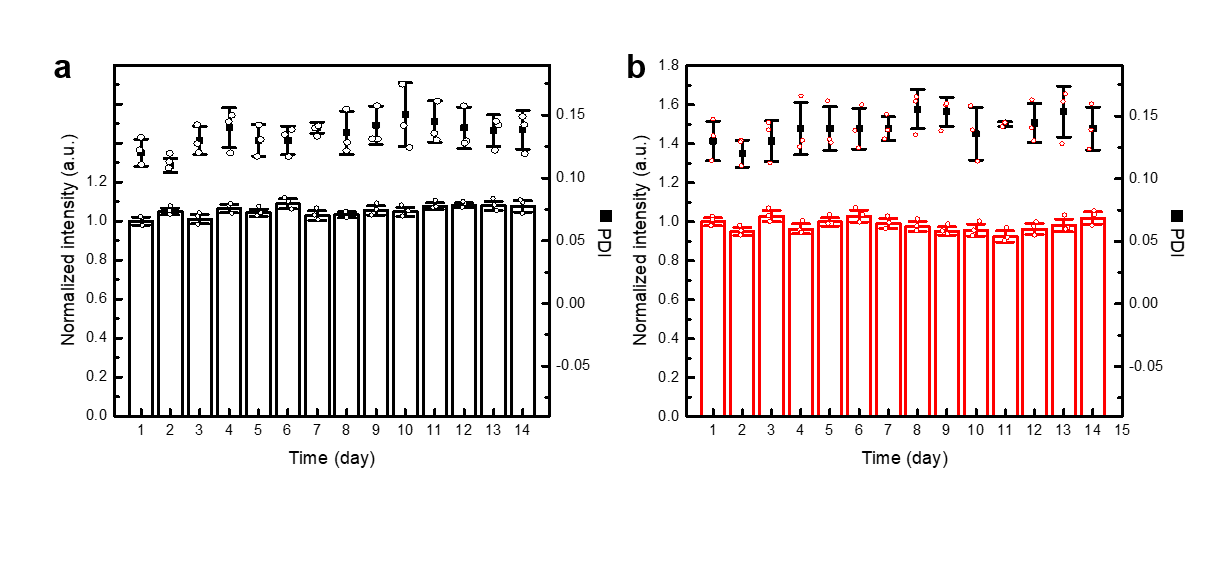


**Supplementary Figure S12.** Two-week stability test of Ab-NPs in a 1 mM PBS solution and a 10 % FBS solution. Time-dependent luminescence intensity and polydispersity index (PDI) of Ab-NP in a) a 1 mM PBS solution and b) a 10 % FBS solution. Data are expressed as mean ± SEM (n = 3, independent experiments) Source data are provided as a Source Data file.


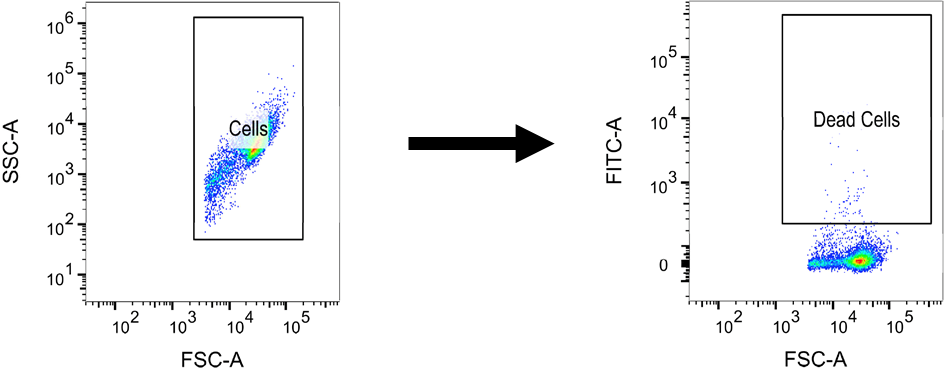


**Supplementary Figure S13.** A representative FACS plots showing the gating strategy. The cell population sorted by FSC-A/SSC-A was subgated by FSC-A/FITC-A to quantify the percentage of SYTOX-Green positive cells, which means dead cells. Source data are provided as a Source Data file.


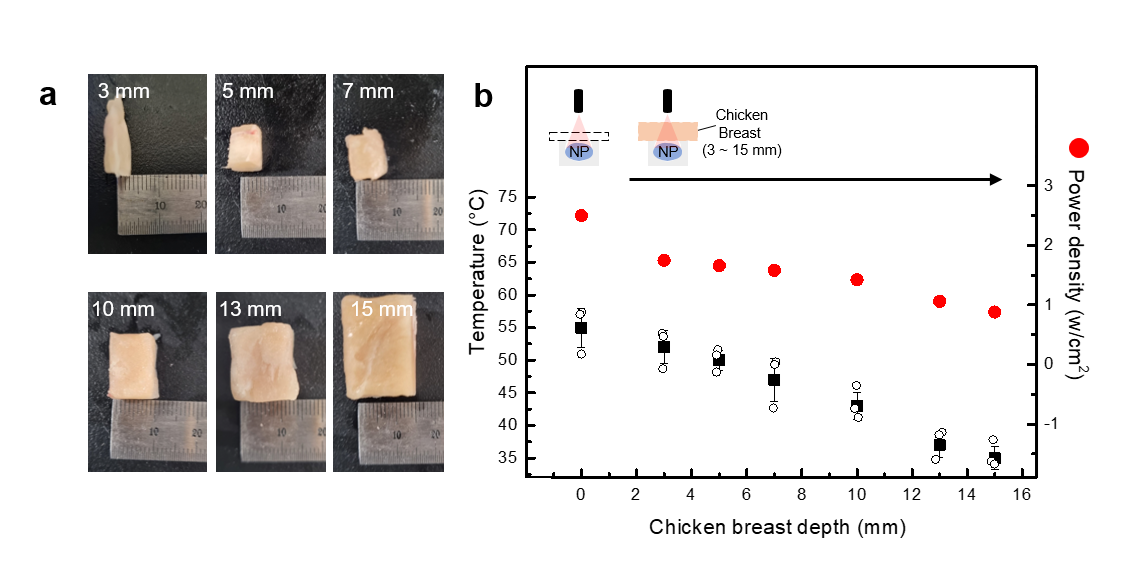


**Supplementary Figure S14.** Temperature and power density profiles with increasing depth in the chicken breast (3, 5, 7, 10, 13, and 15 mm) under 808 nm laser irradiation for 10 min. The transmitted energy density was directly measured under the chicken breast (Model: Gentec Maestro). Data are expressed as mean ± SEM (n = 3, independent experiments) Source data are provided as a Source Data file.


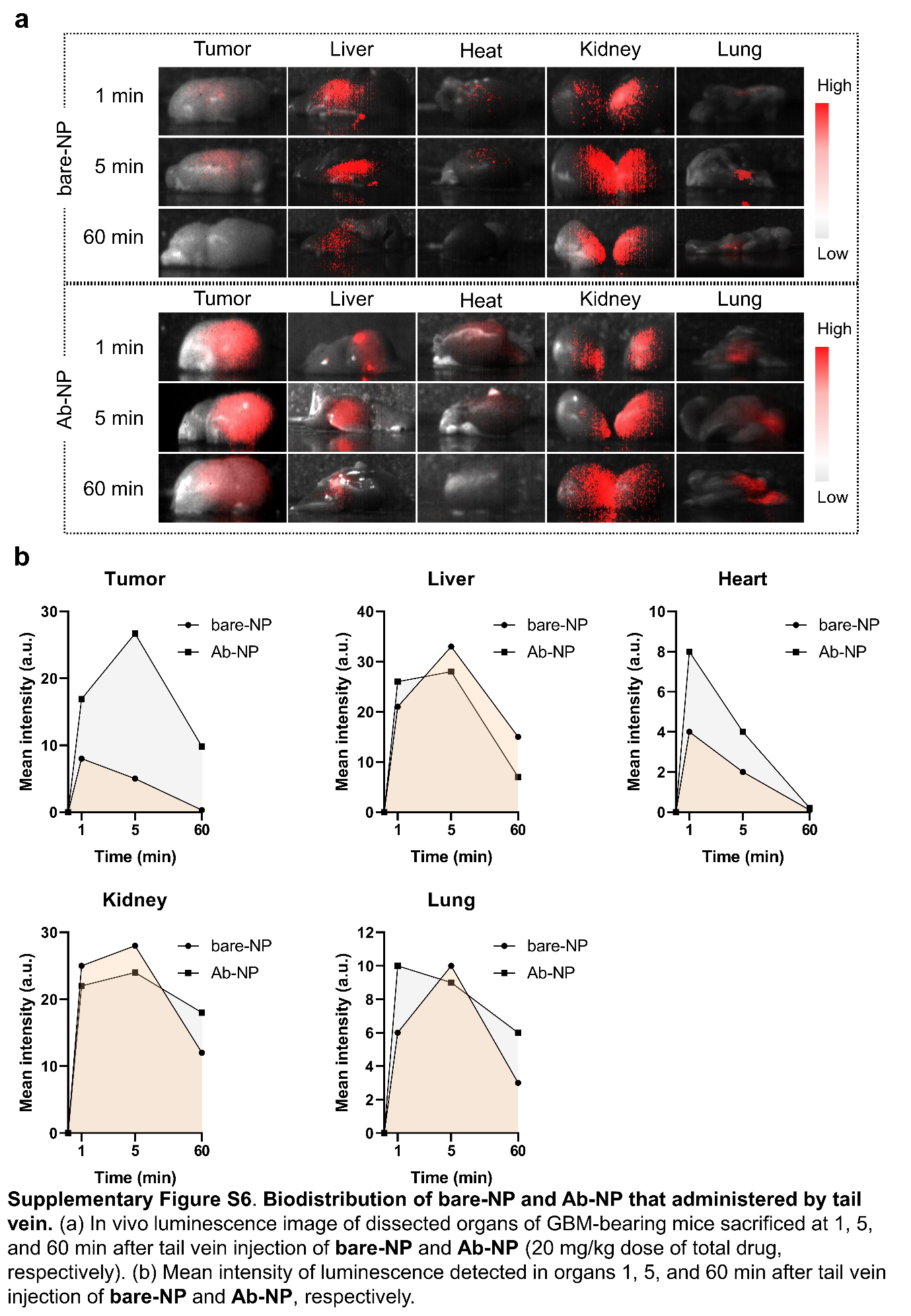


**Supplementary Figure S15**. Biodistribution of bare- and Ab-NPs administered through the tail vein. a) In vivo luminescence images of dissected organs of GBM-bearing mice sacrificed at 1, 5, and 60 min after the tail vein injection of **bare- and Ab-NPs** (20 mg/kg dose of the total drug). b) Mean intensity of luminescence detected in organs 1, 5, and 60 min after the tail vein injection of **bare- and Ab-NPs**. Source data are provided as a Source Data file.


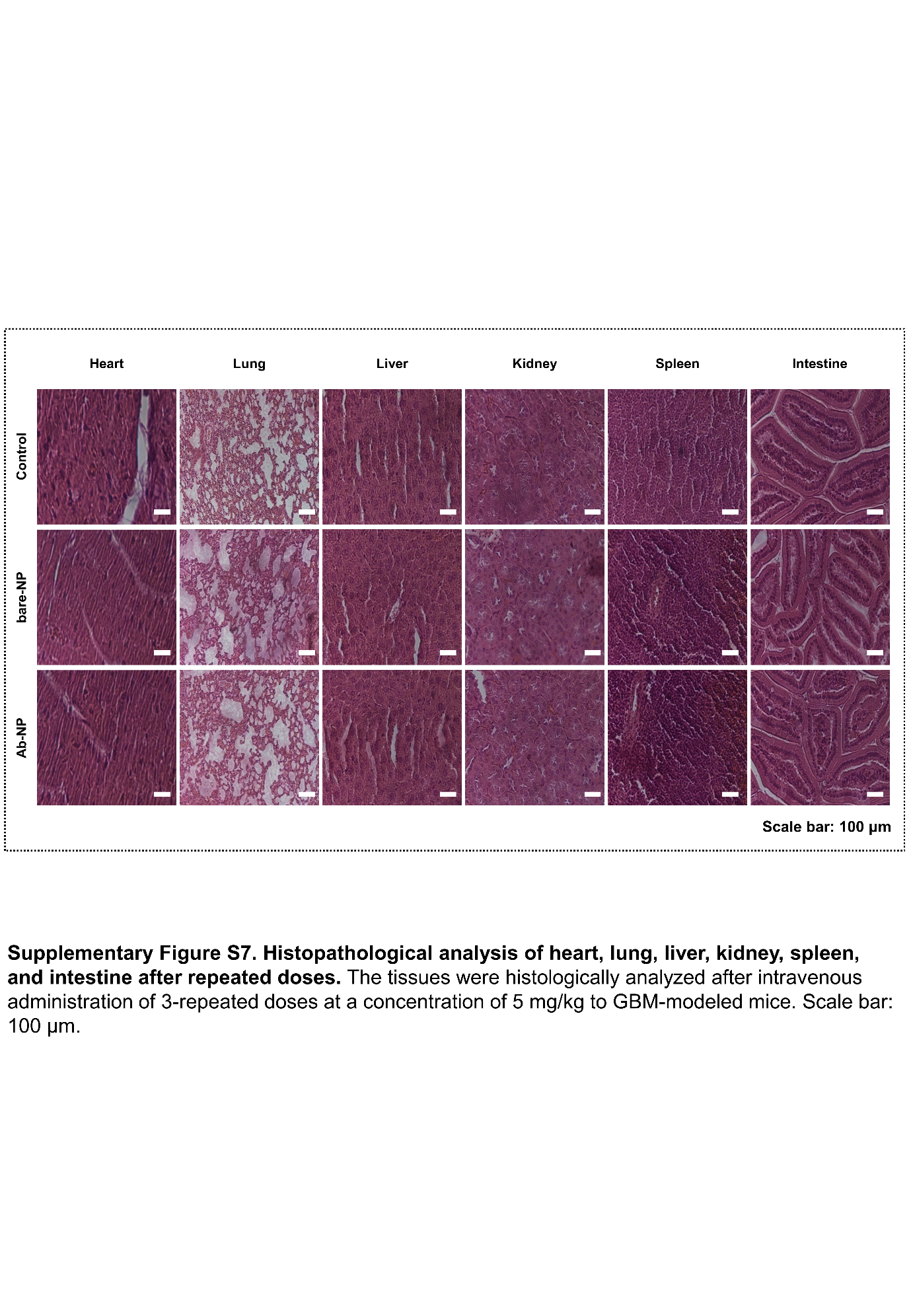


**Supplementary Figure S16.** Histopathological analysis of heart, lung, liver, kidney, spleen, and intestine after repeated doses. The tissues were histologically analyzed after intravenous administration of three repeated doses at a concentration of 5 mg/kg to GBM-modeled mice. Scale bars: 100 μm. Source data are provided as a Source Data file.


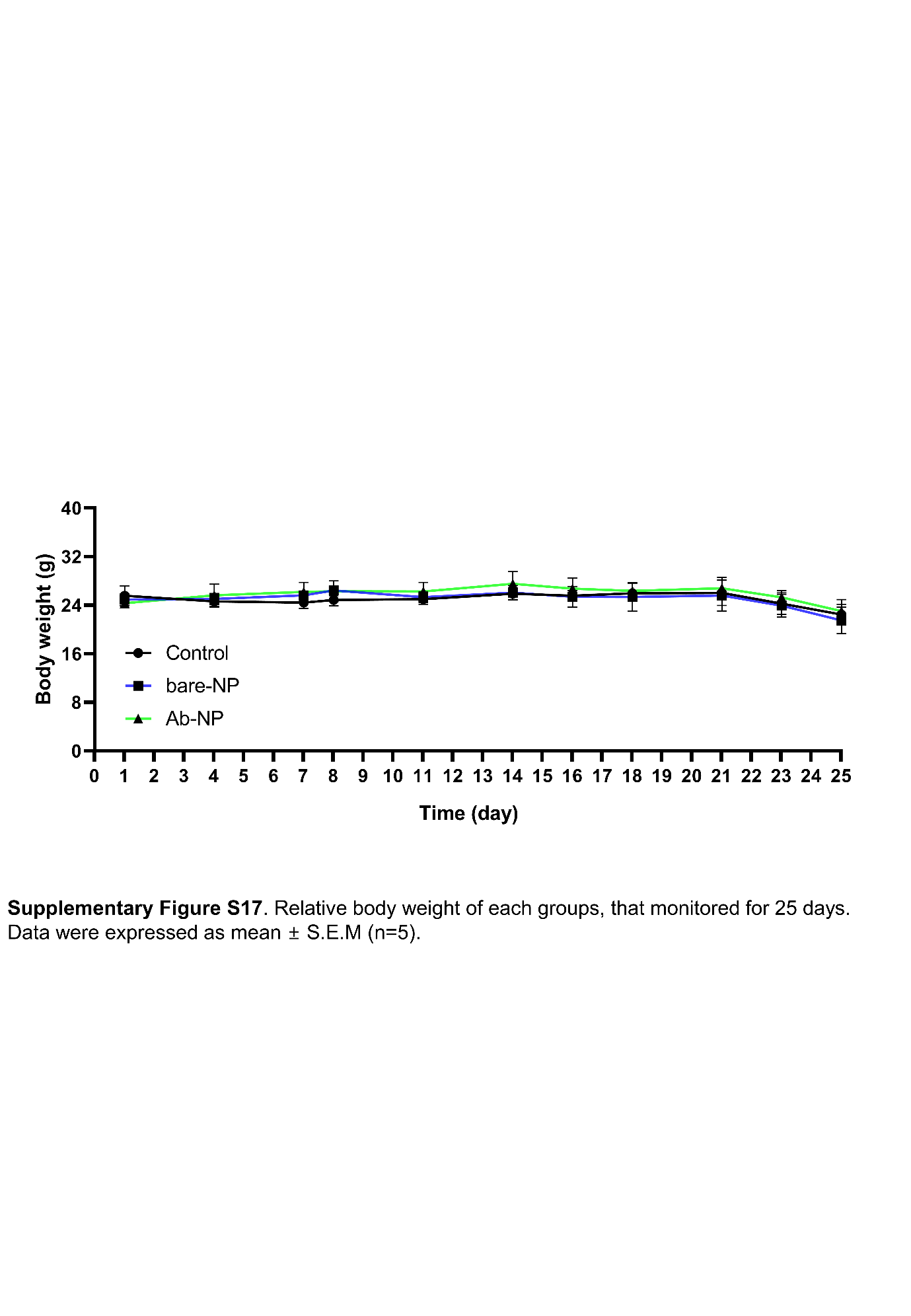


**Supplementary Figure S17**. The relative body weight of mice in each group was monitored for 25 days. Data are expressed as mean ± S.E.M (n = 5 biological independent animals). Source data are provided as a Source Data file.


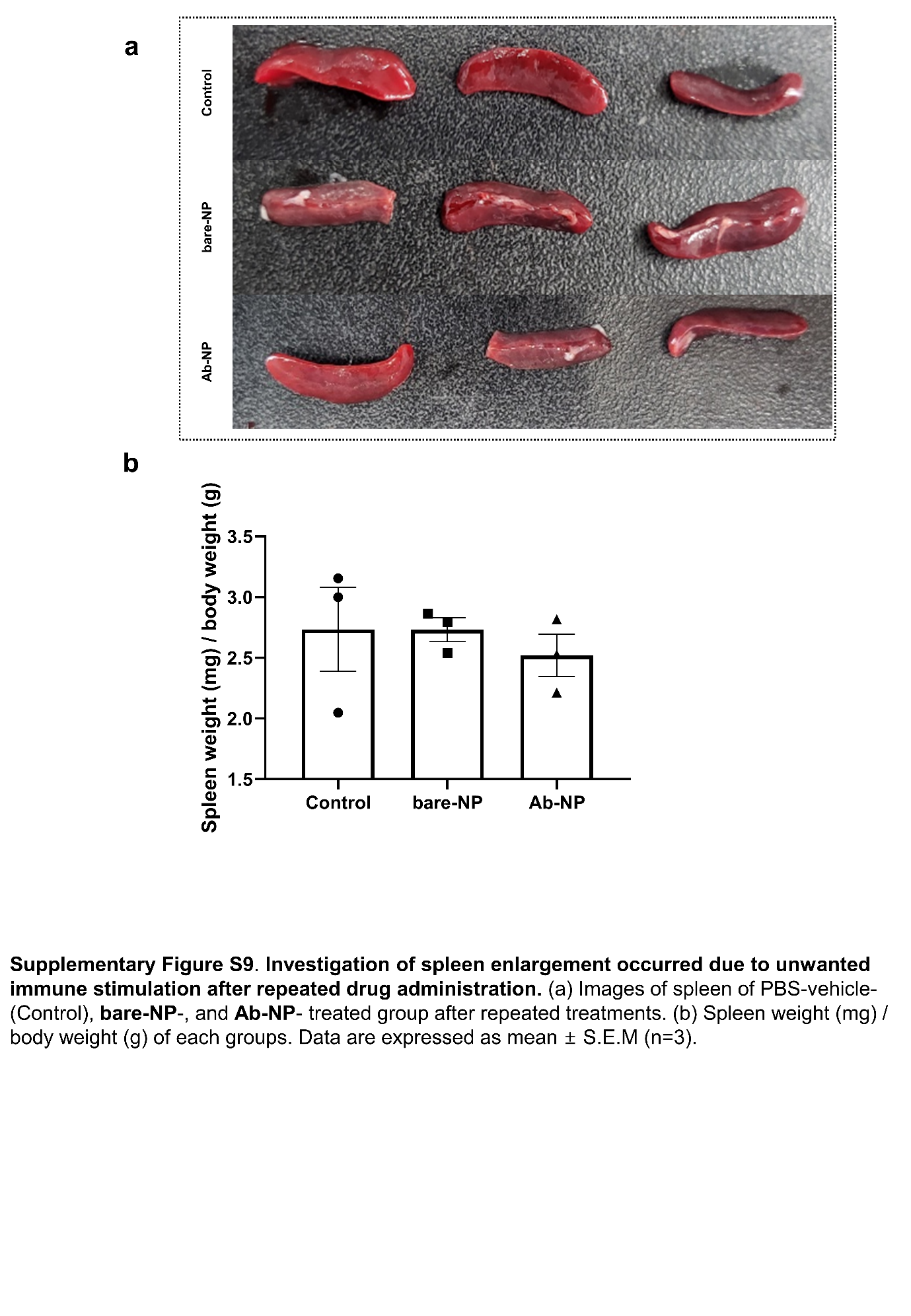


**Supplementary Figure S18.** Investigation of spleen enlargement caused by unwanted immune stimulation after repeated drug administration. a) Images of spleens from groups treated three times with the PBS-vehicle (control), bare NPs, and Ab-NPs. b) Spleen weight (mg) / body weight (g) of each group. Data are expressed as mean ± SEM (n = 3 biological independent animals). Source data are provided as a Source Data file.


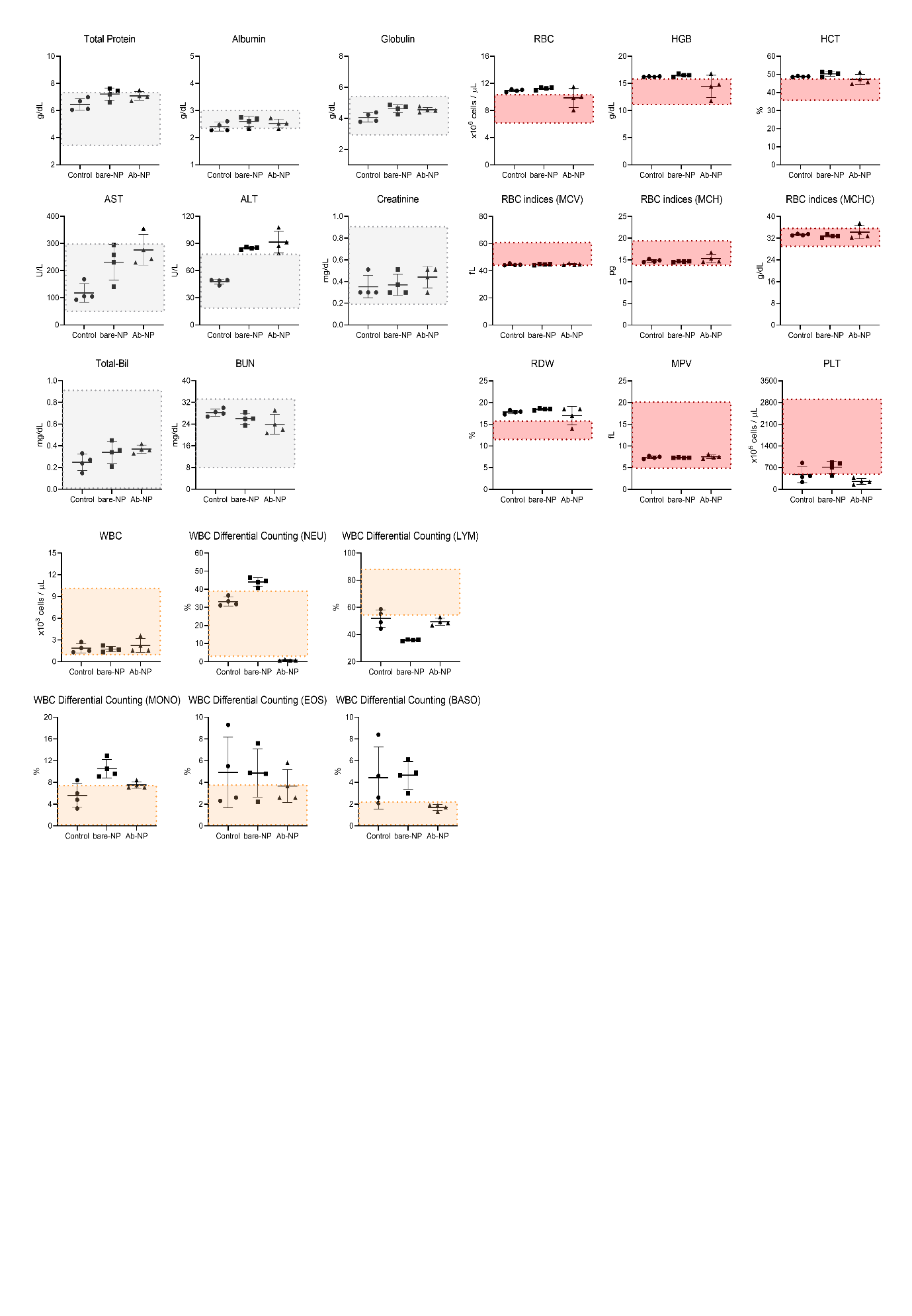


**Supplementary Figure S19.** Biochemistry and CBC tests after intravenous administration three times over one week. The colored boxes indicate the normal range of each index. Data are expressed as mean ± SEM (n = 4 biological independent animals). Source data are provided as a Source Data file.0
