# Supplementary material for: A local water molecular-heating strategy for near-infrared long-lifetime imaging-guided photothermal therapy of glioblastoma
Source: Nat Commun. 2023 May 13;14:2755. doi: 10.1038/s41467-023-38451-3 (PMC10183012; doi:10.1038/s41467-023-38451-3)
Supplement: Supplementary file 2 — Reporting Summary [file 41467_2023_38451_MOESM2_ESM.pdf]

nature portfolio

Corresponding author(s): Joonseok Lee
Dong Yun Lee

Last updated by author(s): Apr 6, 2023

Reporting Summary

Nature Portfolio wishes to improve the reproducibility of the work that we publish. This form provides structure for consistency and transparency in reporting. For further information on Nature Portfolio policies, see our Editorial Policies and the Editorial Policy Checklist.

Statistics

For all statistical analyses, confirm that the following items are present in the figure legend, table legend, main text, or Methods section.

n/a	Confirmed	
☐	☒	The exact sample size (n) for each experimental group/condition, given as a discrete number and unit of measurement
☐	☒	A statement on whether measurements were taken from distinct samples or whether the same sample was measured repeatedly
☒	☐	The statistical test(s) used AND whether they are one- or two-sided<br>*Only common tests should be described solely by name; describe more complex techniques in the Methods section.*
☒	☐	A description of all covariates tested
☒	☐	A description of any assumptions or corrections, such as tests of normality and adjustment for multiple comparisons
☐	☒	A full description of the statistical parameters including central tendency (e.g. means) or other basic estimates (e.g. regression coefficient) AND variation (e.g. standard deviation) or associated estimates of uncertainty (e.g. confidence intervals)
☒	☐	For null hypothesis testing, the test statistic (e.g. F, t, r) with confidence intervals, effect sizes, degrees of freedom and P value noted<br>*Give P values as exact values whenever suitable.*
☒	☐	For Bayesian analysis, information on the choice of priors and Markov chain Monte Carlo settings
☒	☐	For hierarchical and complex designs, identification of the appropriate level for tests and full reporting of outcomes
☒	☐	Estimates of effect sizes (e.g. Cohen's d, Pearson's r), indicating how they were calculated

Our web collection on statistics for biologists contains articles on many of the points above.

Software and code

Policy information about availability of computer code

Data collection	Flow cytometry data were collected using FACS DIVA 8.0.3 software. Microscopy images were collected using Leica Application Suite X 2.0. TEM images were collected by JEN-2100F (JEOL. Ltd). The XRD patterns were recorded by an XRD-7000 diffractometer. A Zetasizer Nano ZSP instrument (Malvern Co., UK) was used to determine the zeta potential. The Fourier transform infrared (FT-IR) spectra were obtained by using an iS10 Fourier transform infrared spectrophotometer (Thermo Fisher scientific Co., USA) A Jasco V-700 UV-Vis spectrophotometer (Jasco, Japan) was used to record the absorbance spectra. The lifetime and images were obtained by the iStar intensified sCMOS camera (Andor) using time-gated imaging technology.
Data analysis	GraphPad Prism 9.5 was used for statistical analysis. Flow cytometry data were analyzed using Flowjo 10.8.1 software. Histology images were processed and analyzed using NDP view 2.8. Sample images and lifetime images were analyzed by using Andor software (Solis 64) and ImageJ program (FlimJ-plugin).

For manuscripts utilizing custom algorithms or software that are central to the research but not yet described in published literature, software must be made available to editors and reviewers. We strongly encourage code deposition in a community repository (e.g. GitHub). See the Nature Portfolio guidelines for submitting code & software for further information.

Data

Policy information about availability of data

All manuscripts must include a data availability statement. This statement should provide the following information, where applicable:

- Accession codes, unique identifiers, or web links for publicly available datasets
- A description of any restrictions on data availability
- For clinical datasets or third party data, please ensure that the statement adheres to our policy

The data which is available upon request, please consider making this data available in a publicly accessible repository, or explain to the editor why this data can only be made available from the authors on request.

Human research participants

Policy information about studies involving human research participants and Sex and Gender in Research.

Reporting on sex and gender	N/A
Population characteristics	N/A
Recruitment	N/A
Ethics oversight	N/A

Note that full information on the approval of the study protocol must also be provided in the manuscript.

Field-specific reporting

Please select the one below that is the best fit for your research. If you are not sure, read the appropriate sections before making your selection.

☒ Life sciences ☐ Behavioural & social sciences ☐ Ecological, evolutionary & environmental sciences

For a reference copy of the document with all sections, see nature.com/documents/nr-reporting-summary-flat.pdf

Life sciences study design

All studies must disclose on these points even when the disclosure is negative.

Sample size	To ensure adequate statistical power and account for system variability, we selected 5 to 8 mice per group for all animal experiments. These sample sizes were derived from at least 3 biological independent experiments, and were deemed sufficient based on our initial experiments with this model. We took into consideration the variability of the system and the ability to detect key outcomes of each experiment analytically. For all nanoparticle, statistic provided figure of sample size was performed at least 3-times independent experiments. And also, sample size was stated in the all figure captions.
Data exclusions	No data were excluded from the analyses.
Replication	At least three replicates were analyzed in each independent experiment to ensure the experimental results were reliable. Biological and technical replicates were considered. At least 2 or 3 independent experiments were performed to validate key data.
Randomization	To ensure unbiased results, experimental animals and samples were randomly assigned to different treatment groups. For brain section work, we selected sections that were closest to the injection site, specifically at 2 mm right lateral and 2 mm posterior from the bregma, which was the orthotopic injection site for glioma cells
Blinding	Blinding was not employed in this study as we did not consider it necessary to mitigate potential subjectivity of the researcher. We applied the same measurements and analysis techniques uniformly to all experimental groups, which minimized the potential for bias

Reporting for specific materials, systems and methods

We require information from authors about some types of materials, experimental systems and methods used in many studies. Here, indicate whether each material, system or method listed is relevant to your study. If you are not sure if a list item applies to your research, read the appropriate section before selecting a response.

Materials & experimental systems

n/a	Involved in the study	
☐	☒	Antibodies
☐	☒	Eukaryotic cell lines
☒	☐	Palaeontology and archaeology
☐	☒	Animals and other organisms
☒	☐	Clinical data
☒	☐	Dual use research of concern

Methods

n/a	Involved in the study	
☒	☐	ChIP-seq
☐	☒	Flow cytometry
☒	☐	MRI-based neuroimaging

Antibodies

Antibodies used	Monoclonal antibodies (e.g. Prominin-1 and anti-EGFR) were purchased from ThermoFisher Scientific and Abcam, respectively. As for the secondary antibody, goat anti-rabbit IgG-H&L Alexa Fluor 488 (ab150077) and goat anti rabbit IgG H&L Alexa Fluor 647 (ab150079) were used and purchased from Abcam.<br>Prominin-1, Invitrogen, Clone TMP4, Dilution 1:100, Catalog # 12-1338-42<br>Anti-EGFR, Abcam, Clone EP38Y, Dilution 1:100, Catalog # ab52894<br>Goat Anti-Rabbit IgG H&L (Alexa Fluor® 488), Clone: IgG2B, Dilution 1:500, Catalog # ab150077<br>Goat Anti-Rabbit IgG H&L (Alexa Fluor® 647), Clone: Polyclonal, Dilution 1:500, Catalog # ab150079
Validation	As for Prominin-1, Purity: Greater than 90%, as determined by SDS-PAGE, Aggregation: Less than 10%, as determined by HPLC. As for anti-EGFR, Purity: Protein G purified.<br>Prominin-1 and anti-EGFR antibodies for immunofluroescence staining applications were well-established and commercialized products that have been extensively tested and validated by the manufacturers as stated on the websites (ThermoFisher Scientific and Abcam)

Eukaryotic cell lines

Policy information about cell lines and Sex and Gender in Research

Cell line source(s)	U87MG human glioma cell line was distributed from Korean Cell Line Bank (seoul, Korea).
Authentication	None of the cell lines used were authenticated.
Mycoplasma contamination	Cell lines were not tested for mycoplasma contamination.
Commonly misidentified lines<br>(See ICLAC register)	No commonly misidentified cell lines were used.

Animals and other research organisms

Policy information about studies involving animals; ARRIVE guidelines recommended for reporting animal research, and Sex and Gender in Research

Laboratory animals	Balb/c nude mice and Balb/c mice of seven-week-old were purchased from Nara-Bio Company, Seoul, Korea. Animals were bred and housed in a standard barrier animal facility at Hanyang University with the light cycle of 14:10, ambient temperature at 22 °C, and relative humidity range between 30-70%. Experimental animals were randomly assigned to into different treatment groups at 6 to 8 weeks old. Experimental and control animals were co-housed. All animal-related experiments were performed in full compliance with animal protocols approved by the Hanyang University Institutional Animal Care and Use Committee (IACUC).<br>Balb/c nude mice and Balb/c mice of seven-week-old were purchased from Nara-Bio Company, Seoul, Korea. Animals were bred and housed in a standard barrier animal facility at Hanyang University with the light cycle of 14:10, ambient temperature at 22 °C, and relative humidity range between 30-70%. Experimental animals were randomly assigned to into different treatment groups at 6 to 8 weeks old. Experimental and control animals were co-housed. All animal-related experiments were performed in full compliance with animal protocols approved by the Hanyang University Institutional Animal Care and Use Committee (IACUC).
Wild animals	The study did not involve wild animals.
Reporting on sex	All animal experiments were conducted on male mice.
Field-collected samples	The study did not involve samples collected from the field.
Ethics oversight	All animals were housed in specific pathogen-free conditions and maintained under the Institutional Animal Care and Use Committee (IACUC: 2021-0108A) at Hanyang University

Note that full information on the approval of the study protocol must also be provided in the manuscript.

Flow Cytometry

Plots

Confirm that:

☒ The axis labels state the marker and fluorochrome used (e.g. CD4-FITC).

☒ The axis scales are clearly visible. Include numbers along axes only for bottom left plot of group (a 'group' is an analysis of identical markers).

☒ All plots are contour plots with outliers or pseudocolor plots.

☒ A numerical value for number of cells or percentage (with statistics) is provided.

Methodology

Sample preparation	1) U87MG cells (80% confluency in 48 well-plate culture dish) were treated with bare-NP or Ab-NP for 10 min. 2) Laser irradiation were applied for 5 and 10 min, respectively, to induce apoptosis. 3) Additional controls was prepared to set up flow cytometer compensation and quadrants. 4) Harvested untreated and treated cells (1 x 10A6 cells/ml). 5) Washed cells twice at room temperature in PBS. 6) Resuspended cells in lX Binding Buffer Solution at a final concentration 1 x lOAG cells/ ml. 7) To each 100 L of cell suspension, added 5 L of Annexin V-DY-634 Conjugate and 5 L of Propidium Iodide Staining Solution. 8) Incubated cells at room temperature for 15 minutes in the dark. 9) Added 400 L of lX Binding Buffer Solution. 10) Analyzed cells by flow cytometry within one hour.
Instrument	FACS CaliburTM; BD Biosciences, Franklin Lakes, NJ
Software	BD FACSdiva software
Cell population abundance	Cell sorting was done in a FACS Aria cytometer (BD Bioscience) and cell purity higher than 97 % was always obtained
Gating strategy	FACS analysis gating was performed on Annexin V/Pi-double-positive apoptotic glioma cells after recording a fixed number of events for all conditions.

☒ Tick this box to confirm that a figure exemplifying the gating strategy is provided in the Supplementary Information.
